# Comparative Analysis of Diagnostic Techniques for Melanoma Detection: A Systematic Review of Diagnostic Test Accuracy Studies and Meta-Analysis

**DOI:** 10.3389/fmed.2021.637069

**Published:** 2021-04-21

**Authors:** Alessia Blundo, Arianna Cignoni, Tommaso Banfi, Gastone Ciuti

**Affiliations:** ^1^The BioRobotics Institute, Scuola Superiore Sant'Anna, Pisa, Italy; ^2^Department of Excellence in Robotics & AI, Scuola Superiore Sant'Anna, Pisa, Italy

**Keywords:** melanoma, diagnosis, non-invasive technique, diagnostic performance, skin cancer, meta-analysis

## Abstract

Melanoma has the highest mortality rate among skin cancers, and early-diagnosis is essential to maximize survival rate. The current procedure for melanoma diagnosis is based on dermoscopy, i.e., a qualitative visual inspection of lesions with intrinsic limited diagnostic reliability and reproducibility. Other non-invasive diagnostic techniques may represent valuable solutions to retrieve additional objective information of a lesion. This review aims to compare the diagnostic performance of non-invasive techniques, alternative to dermoscopy, for melanoma detection in clinical settings. A systematic review of the available literature was performed using PubMed, Scopus and Google scholar databases (2010-September 2020). All human, *in-vivo*, non-invasive studies using techniques, alternative to dermoscopy, for melanoma diagnosis were included with no restriction on the recruited population. The reference standard was histology but dermoscopy was accepted only in case of benign lesions. Attributes of the analyzed studies were compared, and the quality was evaluated using CASP Checklist. For studies in which the investigated technique was implemented as a diagnostic tool (DTA studies), the QUADAS-2 tool was applied. For DTA studies that implemented a melanoma vs. other skin lesions classification task, a meta-analysis was performed reporting the SROC curves. Sixty-two references were included in the review, of which thirty-eight were analyzed using QUADAS-2. Study designs were: clinical trials (13), retrospective studies (10), prospective studies (8), pilot studies (10), multitiered study (1); the remain studies were proof of concept or had undefined study type. Studies were divided in categories based on the physical principle employed by each diagnostic technique. Twenty-nine out of thirty-eight DTA studies were included in the meta-analysis. Heterogeneity of studies' types, testing strategy, and diagnostic task limited the systematic comparison of the techniques. Based on the SROC curves, spectroscopy achieved the best performance in terms of sensitivity (93%, 95% CI 92.8–93.2%) and specificity (85.2%, 95%CI 84.9–85.5%), even though there was high concern regarding robustness of metrics. Reflectance-confocal-microscopy, instead, demonstrated higher robustness and a good diagnostic performance (sensitivity 88.2%, 80.3–93.1%; specificity 65.2%, 55–74.2%). Best practice recommendations were proposed to reduce bias in future DTA studies. Particular attention should be dedicated to widen the use of alternative techniques to conventional dermoscopy.

## Introduction

Malignant melanoma (MM) represents 4% of all cancerous skin lesions and shows the highest crude mortality rate (i.e., 2.9 (1) in USA and 3.6 (1) in Europe, per 100,000 persons, in 2018). To maximize the survival rate, an early diagnosis is essential as current therapeutic options are very effective if promptly adopted (2). Moreover, treatment costs rise with time as the pathology remains untreated, ranging from $4,648 for an *in-situ* melanoma to about $159,808 for a stage IV melanoma (3). The current procedure for the inspection of skin lesions, i.e., dermoscopy, is predominantly qualitative and mainly relies on the visual analysis of each lesion's features. To aid clinicians, a set of standardized diagnostic algorithms are available, such as the 7-points checklist (4) or the ABCDE rule (5). It is known that dermoscopy is dependent upon the examiner's experience and upon the geographical area (6–9). Skvara et al. (8) reported that using the conventional thresholds of the ABCD rule (ABCD score >4.75) and the 7-point checklist (7-point score >2), sensitivity of the ABCD rule was 31.7% with a corresponding specificity of 87.3%, and the sensitivity of the 7-point checklist was 11.1% with a corresponding specificity of 95.2%. Another review (10) reported a 90% sensitivity (95% CI: 80–95%) and 90% specificity (95% CI: 57–98%), achieved by a clinical examination aided by dermoscopy, indicating how dermoscopy can improve clinical examination performance in diagnosing of primary melanoma. Recently, dermoscopy benefitted from the technical evolution of imaging and digital cameras. The use of these new technologies allowed the creation of the so called video-dermoscopy (i.e., digital epiluminescence), paving the way to the application of this diagnostic technique for telemedicine approaches, simplifying the sharing of clinical images, and facilitating follow-up of unclear lesions (11). The current gold standard for melanoma diagnosis is the administration of dermoscopy, followed by a biopsy and subsequent histopathological analysis of the excised tissue. To minimize the risk of misdiagnosis of true melanomas, a significant number of dermoscopically ambiguous lesions are biopsied rising the overall diagnostic costs and time to obtain the final diagnosis. A drawback of dermoscopy is that it allows to obtain only morphological information about a lesion. Beside dermoscopy, other non-invasive diagnostic techniques are available (12, 13). These techniques may be exploited to gain additional information about a lesion, possibly enhancing diagnostic accuracy and reliability. The adoption of different techniques in combination with, or as an alternative to dermoscopy, may increase diagnostic accuracy and clinician's ability to correctly classify skin lesions and assure a prompt melanoma diagnosis in clinical settings.

The aim of this review is to compare the diagnostic accuracy of non-invasive techniques for melanoma detection in clinical setting. Included techniques can be used in combination with or as alternatives to dermoscopy.

## Materials and Methods

**Figure 1:**
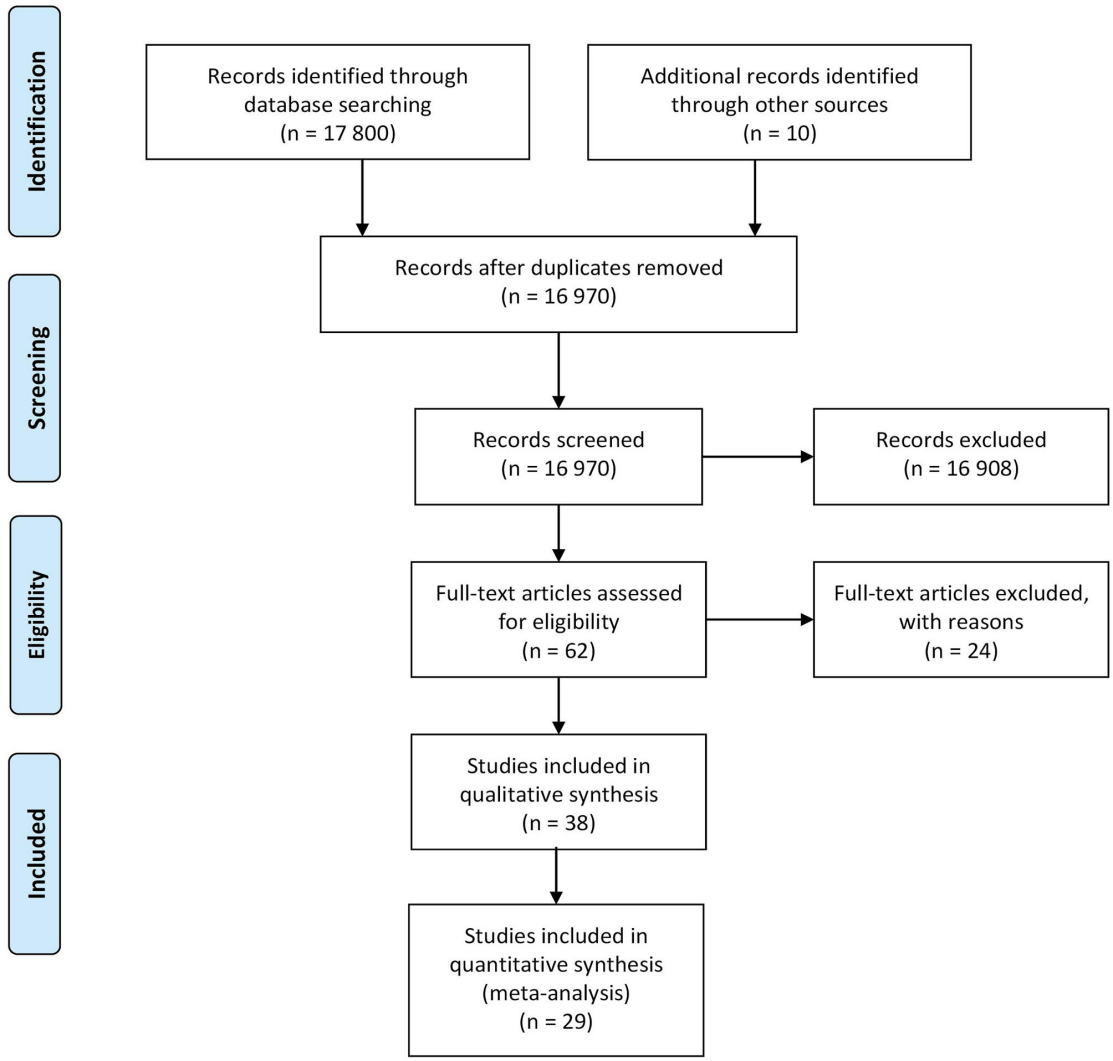
PRISMA diagram outlining the literature review process.

This review of scientific literature followed the methodological guidelines contained in the PRISMA statement (14) for Diagnostic Accuracy Test (DTA) (PROSPERO protocol ID 184123 (15)). A systematic search of the available literature was performed using PubMed, Scopus, and Google scholar databases (time period included 2010-September 2020). The following search query was used: *(“melanoma” OR “skin cancer”) AND (“diagnosis” OR “detection”) AND “non-invasive”*. The PRISMA diagram outlining the literature review process is presented in Figure 1.

### Inclusion Criteria

All studies that use non-invasive techniques alternative to dermoscopy, tested *in-vivo* on humans for melanoma diagnosis, were included with no restriction on age, sex, or ethnicity of the recruited population. The target condition was cutaneous melanoma. No limits on the number of lesions per patient or on the number of patients included in each study were applied. All types of studies were included except for reviews, case control studies and case reports studies. The diagnostic gold standard adopted as reference was histopathology and dermoscopic diagnosis was accepted as a replacement only for benign lesions. Only article written in English were included. The inclusion criteria were applied by AB and AC to the references based on their abstracts to screen their eligibility, while TB reviewed the selection process. Citations were grouped based on the physical principle employed and categorized according to type of non-invasive technique reported by the original study.

### Methodological Analysis

For each included study, experimental design, index test, number of participants and total lesions, inclusion and exclusion criteria, participants' gender and age and reference standard(s) were independently extracted by AB and AC with disagreements solved with discussions. The studies' attributes were reported in Tables 1–3.

**Table 1:** Studies attributes of the 40 included studies exploring optical based techniques for melanoma diagnosis.

**Reference**	**Study design**	**Inclusion criteria**	**Exclusion criteria**	**Sample size (person/lesion)**	**Age (years)**	**Gender**	**Technique**	**Diagnose based on**	**Experince in practice**
**Optical**									
**Optical imaging**									
**Reflectance confocal microscopy**									
(16)	Consecutive case series in two clinics	Melanocytic and non-melanocytic suspicious lesions	Mildly atypical nevi. Lesion under ear and some part of edge of nose and eye	663/710	Median 53 (range 6–90)	309 F, 354 M	RCM	HIST	Expert
(17)	Retrospective study	Superficial nodular lesions	Subcutaneous ones	N/A/140	Mean: 50 (SD = 19.7)	64 M, 76 F	RCM	HIST	Expert
(18)	Consecutive case series	Same as (19)	N/A	62/64	N/A	N/A	RCM	HIST	Expert
(20)	Consecutive case series	Same as (21)	N/A	62/64	N/A	N/A	RCM	HIST	Expert
(22)	Consecutive case series	Same as (19)	Schedule for follow-up or immediate surgical excision	343/343 Excised: 264	54.7 (range 8–89)	136 M, 128 F	RCM + DERM	HIST	Expert
(23)	Retrospective study	Equivocal pigmented lesions excised because changed during follow-up	N/A	70/70	Mean: 39 (F), 40 (M)	32 F, 38 M	RCM and DERM	HIST	Expert
(24)	N/A	Melanocytic lesions	N/A	138/ 138	Median 42 (range 18–78)	90 F, 48 M	RCM	HIST	N/A
(25)	Retrospective web-based study	Same as (21)	N/A	N/A/100	N/A	N/A	RCM	HIST	Expert and recent users
(26)	Retrospective study	Melanocytic lesions with changes in digital dermoscopy	Poor-quality images and lesions exceeding of the system field of view	51/64	Median 42 (range 25–69)	27 F	RCM + DERM	HIST	Expert
(27)	Retrospective study	Lesions excised with suspicious of melanoma	Lesions located on the face and acral sites.	314/333	Median age 50 (range 42–64)	149 M, 184 F	RCM + DERM	HIST	N/A
(28)	Retrospective study	Same as (29)	Same as (27)	389/422	Mean: 47 (range 37–60)	47.8% M	RCM	HIST	Expert
(30)	Retrospective study	Superficial nodular lesions with d > 0.5 cm	Subcutaneous originating lesions	N/A/68	N/A	N/A	RCM + OCT	HIST	Expert
(31)	Prospective analysis	Dark pigmented lesion with clinical-dermoscopic suspicion of melanoma	(1) Lesions with clear-cut features of malignancy (2) Regressive, recurrent or collision tumors (3) Acral lesions (4) Cases with poor quality images	350/370	Median age 45 (29–61)	M 49.1%, F 50.9%	RCM	HIST + Follow up	N/A
**Optical coherence tomography**									
(32)	Pilot study	Same as (33)	N/A	26/26	NA	N/A	HD-OCT (+DERM + RCM)	HIST	Expert
(29)	Retrospective pilot study	Melanocytic suspicious lesions excised	N/A	N/A/45	Mean: 51 (range 25–70)	20 M, 25 F	OCT	HIST	N/A
(34)	N/A	Clinical diagnosed BN or MM	N/A	24/48	N/A	N/A	OCT + DERM	HIST	N/A
(35)	Prospective multicentre study	People scheduled for melanocytic skin lesion excision	Presence of frank ulceration or marked hyperkeratosis	64/93	N/A	N/A	OCT	HIST	Expert
(36)	N/A	Same as (24)	Same as (4, 37)	N/A/39	N/A	N/A	OCT	HIST	Expert
**Multispectral imaging**									
(38)	Two setting clinical trials	Pigmented lesions	N/A	389/639 (UK), 469/581 (AU)	Mean: 44.9 (UK), 50 (AU)	68.6% F (UK), 52% M(AU)	MULTIS	HIST + DERM	Expert
(39)	Proof of concept	Pigmented and vascular lesions	N/A	228/334	N/A	186 F, 39 M	MULTIS	DERM	Expert
(40)	Clinical trial	Same as (38)	N/A	N/A/81	N/A	N/A	MULTIS	N/A	N/A
(37)	Prospective, multicentre, blinded study	Patients with ≥1 pigmented lesions scheduled for biopsy	(1) Same as (21) (2) PL diameter ≥ 22 mm or ≤ 2 mm (3) Anatomic site of PL not accessible to the device (4) Lesion within 1 cm of the eye or on palmar, plantar, or inaccessible site	N/A/1632	Mean 47	F > M	MULTIS	DERMPAT	Expert
(41)	Clinical trial	Melanoma and nevi	N/A	N/A/82	N/A	N/A	MULTIS	HIST + DERM	N/A
(21)	Randomized controlled trial	Suspicious pigmented lesions	No informed consensus	1,297/1,580	Mean 44.6 (SD 16.8)	64% F, 36% M	MULTIS	HIST+ DERM	Expert
(42)	Prospective	Same as (21)	N/A	180/188	Median 43 (range 2–95)	91 F	MULTIS	HIST + DERM	N/A
(43)	N/A	Lesions randomly selected from (37)	N/A	N/A/50	N/A	N/A	MULTIS	DERMPAT	Expert
(44)	Prospective study	Same as (19)	SCC	NA/564	N/A	N.A	MULTIS	HIST	N/A
(45)	NEW device	N/A	N/A	NA/54	N/A	N/A	MULTIS	HIST + DERM	N/A
(46)	Proof of concept	Same as (42)	Other lesions than MM e Nevi	91/100	≥18	N/A	MULTIS	HIST	N/A
(47)	Proof of concept	N/A	N/A	N/A	N/A	N/A	MULTIS	HIST + DERM	Expert
(48)	Prospective study	(1) Lesion warranting further investigation, deemed to be clinically challenging.	(1) Same as (37) (2) Lesions located on other skin condition on the genitalia or mucosa surfaces. (3) Pre-treated or ulcerated lesions (4) Recurrent or metastatic lesions (5) Patients with a Fitzpatrick Phototype > III.	184/209	Patients with melanoma: mean age 62 Patients without melanoma Mean age 48 y (range 17–86)	100 M, 84 F	SPECT, MULTIS + DERM	HIST	EXP
**Optical spectroscopy**									
(33)	Proof of concept	Lesions scheduled for excision	N/A	N/A/50	N/A	N/A	SPECT + EIS	HIST	Expert
(49)	Proof of concept	N/A	N/A	N/A/678	N/A	N/A	SPECT	HIST	N/A
(19)	Clinical trial	Suspicious lesions	(1) Diameter <1 mm (2) Inaccessible site	453/518	Median 61 (range 18–94)	224 M, 229 F	SPECT	HIST + DERM	Expert
(50)	Proof of concept	N/A	N/A	76/137	Median 62 (range 22–93)	71% M, 24% F	SPECT	HIST	N/A
(51)	Feasibility study	N/A	N/A	148/3,072	Mean: 40 (range 2–82)	70% F	SPECT	N/A	N/A
(52)	Preliminary study	database	N/A	N/A/40	N/A	N/A	SPECT	HIST	N/A
(53)	Feasibility study	N/A	N/A	54/56	median 64 (range 44–87)	27 F, 27 M	SPECT	HIST	N/A
(54)	Multicentre, non-randomized clinical trial	(1) Same as (35) (2) Lesions clinically deemed benign	(1) Recent intense UV exposure (2) Pregnancy	787/1,307	Mean age: 61.3	64, 7% M	SPECT	HIST + DERM	N/A
(55)	Prospective study	Suspicious lesions	N/A	52/60	Patients mean age 53, (range 28–87)	29 M	SPECT	HIST	N/A

*The summarized attributes are: (i) study design, (ii) inclusion and exclusion criteria, (iii) sample size (in terms of person/lesion reported), (iv) age of the included population, (v) gender, (vi) technique exploited, (vii) on what the diagnose is based on (e.g., histopathology (HIST), dermoscopy (DERM), pathology (PAT) or Dermatopathology (DERMPAT)), and (viii) experience in practice of operators. N/A, Not Applicable. Techniques: MULTIS (multispectral imaging), SPECT (spectroscopy), RCM (reflectance confocal microscopy), OCT (optical coherence tomography), and DERM (dermoscopy). Yellow rows represent the studies included in the QUADAS analysis (i.e., studies where the specific technique was implemented as a diagnostic tool)*.

**Table 2:** Studies attributes of the 10 included studies exploring EIS based techniques for melanoma diagnosis.

**Reference**	**Study design**	**Inclusion criteria**	**Exclusion criteria**	**Sample size (person/lesion)**	**Age (years)**	**Gender**	**Technique**	**Diagnose based on**	**Experience in practice**
**Skin electrical measurements**									
(56)	Multicentre	Age ≥ 18 years Primary lesions with a suspicion of melanoma scheduled for excision.	(1) Lesions on the sole, palm, under finger and toenails (2) Lesions <2 mm in size (3) Lesions in scars, in beard or mustache	Training set: NA/285 Test set: 183/210	≥18	M/F	EIS using classification algorithm	HIST	Expert
(57)	Multicentre, prospective, non-controlled, non-randomized clinical trial	Same as (56)Maximum eight lesions per patient	(1) Lesions under finger and toenails, in scars or striae (2) Lesions in sites where the electrode could not reach (3) Lesions with abnormal reference areas (4) Crusted lesions	979/1116	≥18	M/F	EIS using classification algorithm	DERMPAT	Expert
(58)	Multicentre, prospective and blinded study	All lesions selected for total excision	Same as (57)	1611/1943	Median 48 (range 18–91)	M/F	EIS	HIST	N/A
(59)	Retrospective descriptive study	Age ≥ 18 with atypical melanocytic lesions	Same as (57)	19/22	Median 53 (range 23–69)	M/F	EIS with ST-SDD	HIST + DERM	N/A
(60)	Observational, prospective, multicentre study	Suspicious melanocytic lesion eligible for short-term sequential digital dermoscopy imaging (SDDI)	Same as (57)	112/160	Median 46 range (23–82)	M/F	EIS paired with SDDI	HIST + DERM	N/A
(61)	Initial evaluation study	N/A	N/A	24/154	N/A	N/A	EIS	N/A	N/A
(62)	*Post-hoc* analysis for retrospective study	Patients of (58) with melanoma lesions	N/A	N/A	N/A	N/A	EIS + DERM	HIST	N/A
(63)	Online survey for trainees	N/A	N/A	-/45	N/A	N/A		N/A	N/A
(64)	Clinical pilot study	Age ≥ 18 years with suspicious skin lesions. Lesions must be accessible to the probe.	(1) Other skin diseases in the same/nearby localization (2) Pregnancy (3) Breastfeeding.	51/59	≥18	M/F	Parelectric spectroscopy	HIST	N/A
(65)	Multitiered study	Clinically suspicious lesions from a previous trial	N/A	-/43	N/A	N/A	EIS	HIST	N/A

*The summarized attributes are: (i) study design, (ii) inclusion and exclusion criteria, (iii) sample size (in terms of person/lesion reported), (iv) age of the included population, (v) gender, (vi) technique exploited, (vii) on what the diagnose is based on (e.g., histopathology (HIST), dermoscopy (DERM), pathology (PAT) or Dermatopathology (DERMPAT)), and (viii) experience in practice of operators. N/A, Not Applicable. Yellow rows represent the studies included in the QUADAS analysis (i.e., studies where the specific technique was implemented as a diagnostic tool). Technique: EIS (skin electrical impedance measurements tomography) and DERM (dermoscopy)*.

**Table 3:** Studies attributes of the 12 included studies exploring thermal based techniques for melanoma diagnosis.

**Reference**	**Study design**	**Inclusion criteria**	**Exclusion criteria**	**Sample size (person/lesion)**	**Age (years)**	**Gender**	**Technique**	**Diagnose based on**	**Experience in practice**
**Thermal measurements**									
(66)	Pilot clinical trial	Patients with pigmented lesion that was suspicious for malignancy and needed to be biopsied	N/A	24/-Showed only two lesions	N/A	N/A	IR-D	HIST	N/A
(67)	Pilot clinical trial	Same as (66)	N/A	35/-	N/A	N/A	IR-D	HIST	N/A
(68)	Patient study	N/A	N/A	N/A	N/A	N/A	IR-D	N/A	N/A
(69)	Pilot patient study	Same as (66)	N/A	37/- Showed only four lesions	N/A	N/A	IR-D	HIST	N/A
(70)	Pilot clinical trial	Same as (66)	N/A	37/- Showed seven lesions	N/A	N/A	IR-D	PAT	N/A
(71)	Unicentral study	N/A	N/A	30/-	N/A	N/A	IR-D + IR-SS	DERMPAT	Experts
(72)	Cross-sectional study	N/A	N/A	102/102	N/A	58%M	IR-D	HIST + DERM	N/A
						42% F			
(73)	Pilot study	N/A	N/A	151/151	N/A	58%M	IR-D	HIST + DERM	N/A
						42%F			
(74)	-	N/A	N/A	85/50	N/A	N/A	IR-SS	HIST	N/A
(75)	Pilot study	N/A	N/A	11/11	≥21	M/F	TCM	DERMPAT + PAT	N/A
(76)	-	N/A	N/A	-/320	N/A	N/A	IR-D + IR-SS	N/A	N/A
(77)	-	Subjects with age ≥ 18	Lesions located on area of injury risk or impossible access	-/41	≥18	N/A	IR-SS + IR-D	HIST	N/A

*The summarized attributes are: (i) study design, (ii) inclusion and exclusion criteria, (iii) sample size (in terms of person/lesion reported), (iv) age of the included population, (v) gender, (vi) technique exploited, (vii) on what the diagnose is based on (e.g., histopathology (HIST), dermoscopy (DERM), pathology (PAT) or Dermatopathology (DERMPAT)), and (viii) experience in practice of operators. N/A, Not Applicable. Yellow rows represent in the QUADAS analysis (i.e., studies where the specific technique was implemented as a diagnostic tool). Techniques: IR dynamic thermal imaging (IR-D), IR steady-state thermal imaging (IR-SS) and thermal conductivity measurements (TCM)*.

To provide a standardized measure of methodological quality of each study, i.e., evaluating criteria such as, the amount of data collected and the appropriateness of data analysis, the CASP Qualitative checklist (78) was employed (excluding point 10 as it is not applicable, Table 4). This checklist was used to compare the quality of the studies within techniques based on different physical principle. For those studies where the specific technique was implemented as a diagnostic tool (i.e., diagnostic results were compared to biopsy results) the assessment of the study's quality was carried on using also the QUADAS-2 tool (79), hence examining bias and applicability of the studies with respect to four separate domains: (i) patient selection, (ii) index test (i.e., diagnostic technique investigated in the study), (iii) reference standard (i.e., the ground truth technique used as reference), and (iv) the patient flow and timing in the study. For each QUADAS2 domain, any concern regarding bias and applicability were scored as “low,” “high,” or “unclear,” based on the information given by the authors in each publication. These results belonging to single studies using the same technique were merged together and were then presented in graphical form (Supplementary Figures 1B,C, 2B,C, 3B,C, 4B,C). Single studies results were presented in the same figures in a table form (Supplementary Figures 1A, 2A, 3A, 4A). Following QUADAS-2 tool guidance, any domain judged at high risk of bias made the whole study considered at high risk of bias. Risk of bias and a concern regarding applicability in patient selection was considered high when only pre-selected patients or patients with lesions with a high concern of melanoma were included in the study. The risk of bias in the index test was considered high in studies where the threshold was selected after the test and a high concern of applicability was considered for studies where the index test was analyzed without all the clinical information or visual examination. A high risk of bias in flow and timing was reported for studies where different reference standard were used. Correlational and feasibility studies were excluded from this analysis. Risk of bias assessment was independently performed by AB and AC, with disagreements solved with discussions.

**Table 4:** CASP Checklist for each study included in this review: Yes (Y), Unclear (U), Can't tell (N/A).

**Reference**	**Is there a clear statement of the aims of research**	**Is qualitative methodology appropriate**	**Is the study design appropriate to address the aim**	**Was the recruitment strategy appropriate**	**Was the data collected in a way to address the aim**	**Has the relationship between researcher and subjects been adequately considered**	**Have ethical issues been taken into consideration**	**Was the data analysis sufficiently rigorous**	**Is there a clear statement of findings**
**Optical Imaging**									
**Reflectance confocal microscopy**									
(16)	Y	Y	Y	U	U	N/A	Y	Y	Y
(17)	Y	Y	Y	U	Y	N/A	Y	U	Y
(18)	Y	Y	Y	U	Y	N/A	N/A	Y	Y
(20)	Y	Y	Y	Y	Y	N/A	Y	Y	Y
(22)	Y	Y	Y	Y	Y	N/A	N/A	Y	Y
(23)	Y	Y	Y	Y	Y	N/A	N/A	Y	Y
(24)	Y	Y	Y	Y	Y	N/A	Y	Y	Y
(25)	Y	Y	Y	Y	Y	N/A	Y	U	Y
(26)	Y	Y	Y	Y	Y	N/A	Y	U	Y
(27)	Y	Y	Y	Y	Y	N/A	N/A	Y	Y
(28)	Y	Y	Y	Y	Y	N/A	Y	Y	Y
(30)	Y	Y	Y	Y	Y	N/A	Y	U	Y
(31)	Y	Y	Y	Y	Y	N/A	Y	Y	Y
**Optical coherence tomography**									
(32)	Y	Y	Y	U	Y	N/A	Y	U	U
(29)	Y	Y	Y	N/A	Y	N/A	Y	Y	Y
(34)	U	Y	Y	N/A	Y	N/A	Y	U	Y
(35)	Y	Y	Y	U	Y	N/A	Y	Y	Y
(36)	Y	Y	U	U	Y	N/A	Y	U	Y
**Multispectral imaging**									
(38)	Y	Y	Y	Y	Y	N/A	Y	Y	Y
(39)	U	U	Y	U	Y	N/A	Y	U	U
(40)	U	Y	Y	U	U	N/A	Y	U	U
(37)	Y	Y	Y	Y	Y	Y	N/A	Y	Y
(41)	Y	Y	Y	U	U	N/A	N/A	U	U
(21)	Y	Y	Y	Y	Y	N/A	Y	Y	Y
(42)	Y	Y	Y	Y	Y	N/A	N/A	Y	Y
(43)	Y	Y	Y	Y	Y	N/A	Y	Y	Y
(44)	Y	Y	Y	U	Y	N/A	Y	Y	Y
(45)	Y	Y	Y	U	Y	N/A	Y	Y	Y
(46)	Y	N/A	Y	U	Y	N/A	Y	U	Y
(47)	U	Y	Y	U	U	N/A	Y	U	U
(48)	Y	Y	Y	U	Y	N/A	Y	U	Y
**Spectral imaging**									
(33)	Y	Y	Y	U	Y	N/A	Y	U	Y
(49)	Y	Y	Y	U	Y	N/A	N/A	Y	Y
(19)	U	Y	Y	Y	Y	N/A	Y	Y	Y
(50)	Y	Y	Y	U	Y	N/A	Y	Y	Y
(51)	Y	Y	Y	U	Y	N/A	N/A	U	Y
(52)	Y	Y	Y	U	U	N/A	N/A	U	Y
(53)	Y	Y	Y	U	Y	N/A	Y	Y	Y
(54)	Y	Y	Y	Y	Y	N/A	Y	Y	Y
(55)	Y	Y	U	U	Y	N/A	Y	Y	Y
**Skin electrical measurements**									
(56)	Y	Y	Y	Y	Y	N/A	Y	U	U
(57)	Y	Y	U	Y	Y	N/A	Y	U	Y
(58)	Y	Y	Y	Y	Y	N/A	Y	Y	Y
(59)	Y	Y	Y	Y	Y	N/A	Y	U	Y
(60)	Y	Y	Y	Y	Y	N/A	Y	U	Y
(61)	Y	Y	U	N/A	N/A	N/A	N/A	U	U
(62)	Y	Y	Y	U	Y	N/A	Y	U	Y
(63)	Y	Y	Y	N/A	N/A	N/A	N/A	Y	Y
(64)	Y	Y	Y	Y	Y	N/A	Y	Y	U
(65)	Y	U	U	U	Y	N/A	N/A	Y	Y
**Thermal measurements**									
(66)	U	Y	Y	U	Y	N/A	Y	U	Y
(67)	Y	Y	Y	U	Y	N/A	Y	U	Y
(68)	Y	Y	Y	N/A	U	N/A	N/A	U	U
(69)	Y	Y	Y	U	Y	N/A	N/A	U	Y
(70)	Y	Y	Y	U	Y	N/A	Y	U	U
(71)	Y	Y	U	N/A	N/A	N/A	N/A	U	U
(72)	Y	Y	Y	U	Y	N/A	Y	Y	U
(73)	Y	Y	U	N/A	Y	N/A	Y	U	U
(74)	Y	Y	U	U	U	N/A	Y	U	U
(75)	Y	Y	Y	Y	Y	N/A	Y	Y	Y
(76)	Y	Y	U	U	U	N/A	N/A	U	U
(77)	Y	Y	U	Y	U	N/A	Y	U	U

*Yellow rows represent the studies included in the QUADAS analysis (i.e., studies where the specific technique was implemented as a diagnostic tool)*.

For those studies aimed to report diagnostic performance of a technique, and thus, for those studies included in the QUADAS-2 analysis, the diagnostic accuracy of the reported technique was compared. A confusion matrix was filled for studies that reported the True Positive (TP), False Negative (FN), True Negative (TN), and False Positive (FP) values. TP was considered a diagnosis of melanoma/malignant lesions using the index test confirmed by histopathological examination. TN was considered a diagnosis of banal nevi or other type of benign lesion confirmed by the reference standard. FP was considered a diagnosis of melanoma/malignant lesion by the index test confirmed to be a banal nevi or other benign skin lesion by the reference standard. FN was considered a diagnosis of banal nevi or benign non-melanoma skin lesion by index test confirmed to be a melanoma/malignant lesion by the reference standard. A meta-analysis of DTA studies was conducted using interactive web-based tool MetaDATA (80, 81). Here, starting from the confusion matrix, sensitivity, and specificity per-lesion (i.e., computed on the number of lesions included in the study) of the technique investigated by each study were computed. For both metrics, the 95% confidence interval (CI) was calculated using the Clopper Pearson method (82). To provide a compact representation of both quality and diagnostic performance metrics of reviewed studies, summary receiver operating characteristics (SROC) curves were drawn, and are depicted in **Figure 3**. Indicators of quality included in the SROC plots were assessed using QUADAS-2 (i.e., overall risk of bias and overall concern regarding applicability). The SROC curves show also a 95% CI region. Only studies that reported the TP, FN, TN, FP values were included. Studies in which the classification task was framed differently from a binary classification between melanoma vs. other benign skin lesions were excluded from the meta-analysis as this inhomogeneity prevented direct comparison of diagnostic performance.

The sensitivity and specificity (paired with their CIs) for each study that reported the aforementioned values were also detailed into a Forest Plot. These information are available in the Supplementary Materials (Supplementary Figures 5–7). In the same figures, DTA studies results excluded from the meta-analysis were reported for completeness.

## Results

The database search yielded a total of 17,800 papers of which 16,970 were unique. After the application of the inclusion and exclusion criteria, 62 papers were included in the review, of which 38 (61.3%) papers targeted the evaluation of the diagnostic performance of a technique and were included in the qualitative analysis (i.e., were considered to be DTA studies and were included in the QUADAS-2 analysis and performance comparison). Starting from the initial pool of raw dataset (i.e., 16,970 studies), the majority of studies (99.6%) were excluded based on their abstract since they were non-*in-vivo* human studies, or did not report the index test in a clinical or pre-clinical setting, or did not include melanoma lesions in their dataset or the reported reference standard was dermoscopy without histopathology results for cancerous lesions. 29 (76.3%) studies of the already selected DTA study list were included in the meta-analysis, indeed, 4 studies (10.5%) were excluded from meta-analysis due to the definition of the classification task into a malignant vs. benign classification instead of the targeted melanoma vs. benign. 5 studies (13.2%) were excluded from the meta-analysis due to the absence of raw values of TN, TP, FN, FP. The PRISMA diagram outlining the literature review process is shown in Figure 1.

**Figure 2:**
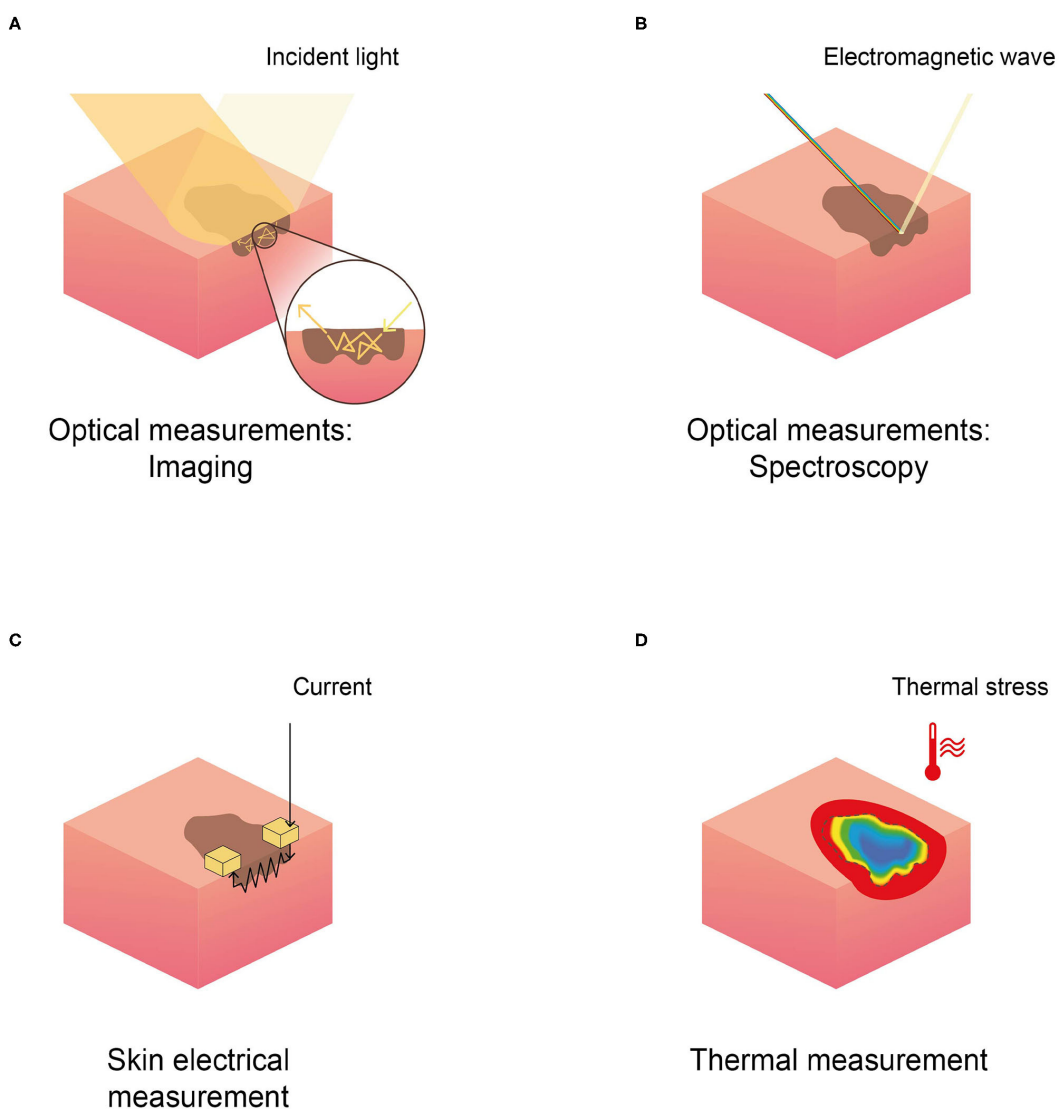
Schematic representation of the physical principles behind different techniques in skin cancer detection, reported in the selected literature. **(A)** Optical imaging, **(B)** optical spectroscopy, **(C)** skin electrical measurement (EIS), and **(D)** thermal measurement.

The general methodological characteristics of the 62 included papers were reported in Tables 1–3; namely we reported study population inclusion and exclusion criteria, sample size (both for patients and lesions), average age, gender distribution, type of study, index test, and reference standard. Studies were grouped based on the physical principle exploited by the non-invasive technique reported: (1) *optical*, both imaging and spectroscopy; (2) *electrical*; and (3) *thermal*. Figure 2 depicts a schematic representation of the physical principles analyzed in this review.

### Optical Measurements

#### Optical Imaging

Three different optical imaging techniques for melanoma diagnosis were found: (i) reflectance confocal microscopy (RCM); (ii) multispectral imaging (MI); and (iii) optical coherence tomography (OCT).

Reliable correlates for epidermal and junctional histological features, useful for diagnostic purposes, were identifiable using RCM imaging (18, 20, 24, 25, 30). Four melanoma scoring algorithms based on these features were validated in literature (18, 27, 83, 84). In Borsari et al. (27) the diagnostic score combines dermoscopy and RCM, while the rest relied exclusively on confocal data. The performances of the four scoring systems have been compared retrospectively by Pampena et al. (28), using different thresholds (i.e., number of features that a lesion presented to be considered melanoma using the algorithm) to assess if a lesion belonged to the melanoma class, suggesting that mixed criteria may be the best solution in reducing false positive rate. Another algorithm based on a two-step model was proposed in Guitera et al. (16). RCM image-based diagnosis is user's dependent and experienced users achieve higher sensitivity than novice users (91 vs. 84.8%), even if the specificity was similar (80 vs. 77.9%) (25). RCM used complementarily to dermoscopy, can increase accuracy in melanoma detection (22, 23, 26) and hence may reduce unnecessary biopsies (22, 23). Moreover, a reduction in the number needed to excise (NNE) for melanoma in dermoscopy compared with RCM was reported by Longo et al. (31). The NNE was 2.9 with clinical-dermoscopy alone and dropped to 1.5 thanks to RCM integration, leading to a 60.6% reduction of unnecessary biopsies and to a sensitivity of 98.1%. RCM may be useful also in the diagnosis of nodular lesions (17).

Different MI systems were found in literature, including two commercial devices, i.e., *SiaScope* (Astron Clinica and Limited, UK), and *MelaFind*^®^ (MELA Sciences, Irvington, NY). The appropriateness of *SiaScope* in improving accuracy of referrals in primary care setting is still under investigation (21, 38, 42), but Sguros et al. (42) proposed to use the device as an additional tool in the hands of less experienced users. *MelaFind*^®^ was validated in aiding dermatologists to provide a more accurate biopsy decision (37, 43), increasing specificity and sensitivity. The use of the multispectral imaging camera *Nuance EX* (CRi, USA) is reported in three studies (40, 41, 85). More recently, a multispectral imaging device based on LED illuminators, capable of sensing texture information of the lesions, have been proposed as a screening tool to assist physician's decision (44, 45, 47). Finally, the diagnostic utility of LED-based hyperspectral imaging (exploiting 21 wavelengths) in combination with machine learning was demonstrated.

5 papers (29, 32, 34–36) investigated OCT. 4 studies (32, 34, 36) reported the correlation between OCT and histological features and only one (35) validated OCT as a diagnostic tool to differentiate cutaneous melanoma and benign melanocytic lesions. All the studies employed the *SkinTell*^®^ (Agfa Healthcare, Mortesel, Belgium) high-definition OCT device except one study (36) that employed the *Vivosight OCT Scanner* (Michelson Diagnostic, Orpington, U.K.).

#### Optical Spectroscopy

Three different spectroscopy techniques were found in literature: (i) Raman spectroscopy (RS); (ii) diffuse reflectance spectroscopy (DRS); and (iii) fluorescence spectroscopy (FS).

The majority of studies investigated the performance of DRS (33, 49, 50, 52, 54). Lim et al. (50) reported the combination of DRS with other spectroscopic methods (RS, Laser-induced-FS), and Bodén et al. (33) the combination of DRS with skin impedance spectroscopy. Only two studies (19, 55) reported the performance of RS, while no studies mentioned performance of FS alone. A study reported a prototype of a RS-AF system (53). Lui et al. (19) showed that classification based on RS is not influenced by lesions location and also suggested different optimized wavebands for different classification tasks (e.g., cancer and precancerous vs. benign). Only one study (54) reported the performance of an investigational EES device, i.e., *Dermasensor*™ (DermaSensor, Inc, Miami, FL). The remaining studies used non-commercial tools. 4 studies (19, 33, 50) highlighted the need of reference measurements of healthy skin to process and analyse spectral data. All the studies used a binary classification output and exploited automatic analysis and classification. The latest study (55) reported a reduction of the number needed to treat for melanoma diagnosis from 8.6 to 4.1 when dermatologists followed the RS model recommendation for biopsy. A recent study (48) compared the sensitivity and specificity of different devices exploiting non-invasive imaging techniques (i.e., *MelaFind*^®^, *Versiante Aura*™ and *Fotofinder*^®^) in melanoma diagnosis, over a total of 209 lesions. The outcomes suggested that these techniques could assist but not replace clinical decision making.

### Skin Electrical Measurements

Electrical impedance spectroscopy (56–63) (EIS) is the leading technique found in literature that involves the measurements of skin electrical properties. For the EIS measurements, the majority of the studies employed the *Nevisense* system (56–60, 62, 63) (SCIBASE AB, Stockholm, Sweden), while only one study (61) used the *Dermasense* system. Three studies used the *Nevisense* to understand its efficacy (58, 62, 63) and safety (58), comparing the diagnostic performance of its decisional score system with the ABCD rule and the 7-point checklist. Gilou et al. (61) collected only two measurements on one melanoma among their data. They compared these measurements with the one of clear skin patches, using paired *t*-test. In two studies (59, 60), the authors paired the *Nevisense* with the short-term digital dermoscopy imaging (SDDI), a follow-up procedure where each lesion is checked after 3 months (i.e., *t* = 3) from the first visit (i.e., *t* = 0). While Rocha et al. (60) concluded that EIS could avoid the need for follow-up in 46.9% of suspicious benign lesions included in the study, Ceder et al. (59) affirmed that no additional malignant lesions were found with EIS at *t* = 3, during follow-up procedure. A study (65) detailing the performance of a multitiered system of decision support system reported that the inclusion of the EIS score in clinical decision making led to a reduction in the number of unneeded biopsies and that the amount of the reduction depends on a clinician's experience, i.e., 14.8% for resident, 16.8% for midlevel, and 16% for practicing dermatologist. More recently, a paraelectric spectroscopy technology has been used for skin cancer application in a correlational study (64).

### Thermal Measurements

Skin thermal properties depends on tissue metabolic activity that in turn is significantly different among benign and malignant lesions. Thermal imaging (66–77) is the leading technique investigated in literature. Thermal cameras were used to obtain skin lesion features at steady-state (71, 74, 77) and in dynamic thermal conditions (66–70, 72–77). In steady-state studies, thermal images were used to obtain temperature features of the investigated lesion, such as, pixels temperature profiles (74) and temperature difference between several type of lesions and the healthy surrounding skin (71). Some authors suggested that the application of a cooling stress is essential to highlight malignancy: indeed, the thermal recovery of the lesion over time differs between malignant and benign tissues. To guarantee a stable measurement system, some authors implemented a data pre-processing pipeline to limit motion artifacts within the recovery phase (66–70, 72, 73). In five studies (66–70), preliminary results of temperature recovery profiles recorded from 3 melanomas and 34 benign lesions (with respect to the surrounding skin) were presented. Godoy et al. (72, 73) added to this pipeline two different decisional algorithms to enable the automatic classification of a lesion (melanoma vs. other benign skin lesions). In Magalhaes et al. (76, 77) a different cooling and processing pipeline was implemented to extract thermal features from steady-state and dynamic imaging to fed machine learning algorithms for different classification tasks.

A recent approach (75), used punctual temperature measurements to compute the effective conductivity of a skin lesion, highlighting significant differences between measurements of invasive and *in-situ* melanoma.

### Studies Evaluation

Studies generally scored from moderate to unclear quality following the CASP checklist (Table 4). Optical based studies achieved higher quality with respect to other techniques. Thermal based studies scored the lowest quality based on the CASP checklist, indeed only few studies reported sufficiently rigorous data analysis (72, 75) and a clear statement of findings (66–70, 75). Among the various analyzed techniques, optical ones are the most widespread in literature, indicating how these techniques are more consolidated and validated with respect to novel techniques, such as EIS and the thermal based ones.

For 61.3% of included studies (38 over 62 studies), the evaluation of risk of bias and concerns regarding applicability was performed and results were presented in Supplementary Figures 1–4; single study quality assessment using QUADAS-2 tool is reported in the same figures, panel (**A**). Proportions of studies with low, high, unclear risk of bias and concern regarding applicability for each domain of non-invasive techniques, grouped with respect of the physical principle, are visually summarized in Supplementary Figures 1B,C (optical imaging); Supplementary Figures 2B,C (optical spectroscopy); Supplementary Figures 3B,C (EIS); and **Figures 4B,C** (thermal).

32 studies out of 38 (84.2%) presented a high risks of bias, 5 studies (17, 26, 50, 55, 63) (13.1%) presented an unclear overall risk of bias, while only one study (58) (2.6%) presented a low overall risk of bias. A similar trend was reported also for concern regarding applicability, where the majority of studies (24, 63.2%) scored a high concern and 10 studies (17, 24, 28, 35, 36, 38, 54, 59, 71, 74, 76) (26.3%) an unclear one. Only 4 studies (21, 38, 42, 72) (10.5%) reported low concerns regarding applicability.

Patient recruitment was mostly performed including dermoscopic pre-selection of suspicious lesions, leading to high risk of bias and concern regarding applicability in patient selection domain. Risk of bias, with respect to flow and timing, was rated high in 15 studies (39.5%, the majority of them exploited thermal and multispectral analysis) since not all patients received the same reference standard and/or not all patients were included in the analysis (e.g., some studies excluded dermoscopically benign lesions from follow up and analysis). Six studies (16, 25, 43, 52, 63, 65) (15.6%) reported interpretation of index tests evaluated remotely without patient analysis or blinding to clinically relevant information, raising concerns regarding the applicability of the index tests in a clinical setting. Sometimes a diagnostic threshold was specified after the diagnostic task itself, introducing a possible bias into the index test (33, 40, 72, 73).

**Figure 3:**
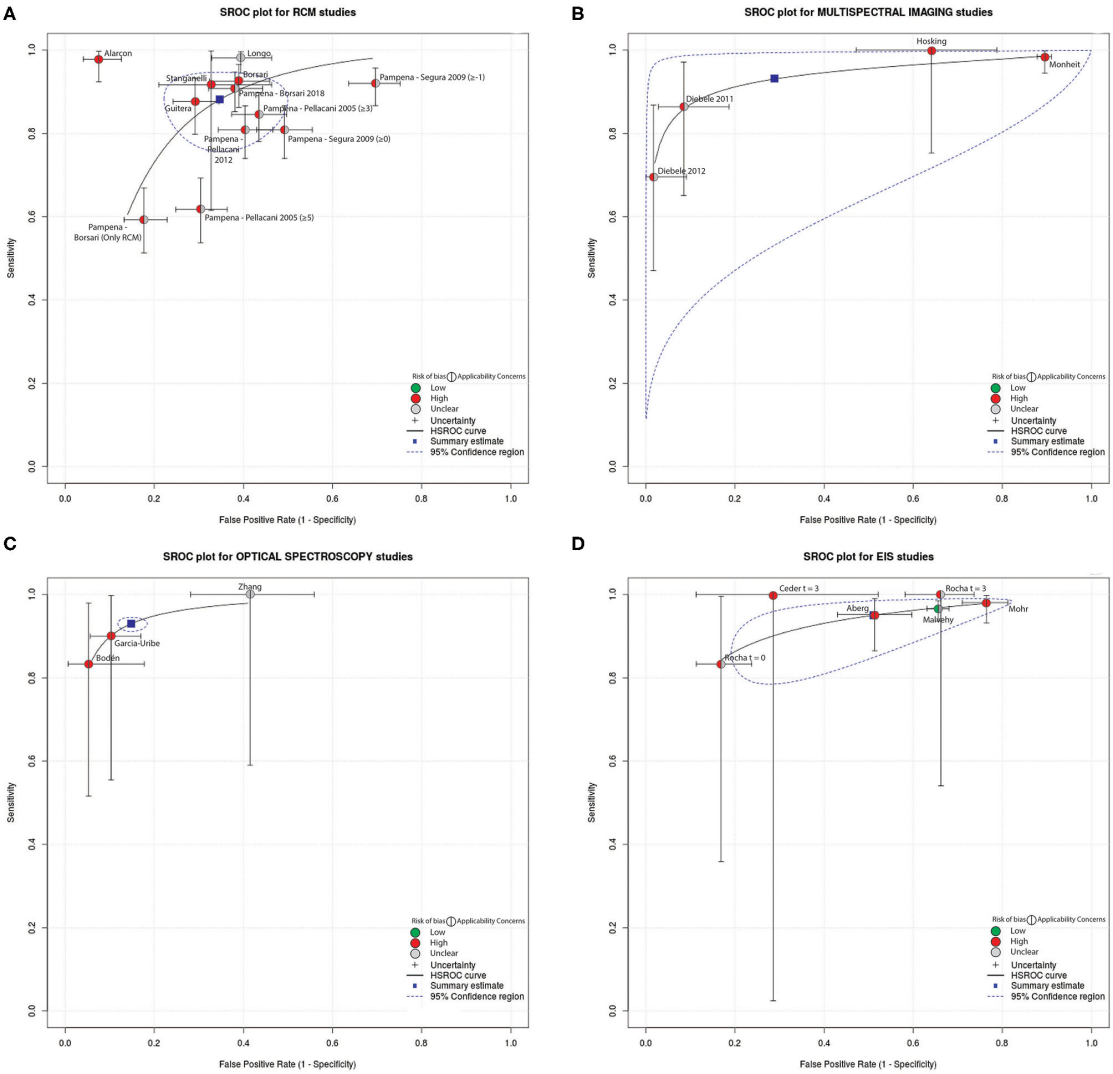
Summary received operating characteristics (SROC) curves which displays the results from the meta-analysis with indicators of quality assessed using QUADAS-2 (i.e., overall risk of bias and overall concern regarding applicability). The curves reported also the 95% Confidence region. Included studies were divided based on the employed technique: **(A)** RCM, **(B)** multispectral imaging, **(C)** spectroscopy, and **(D)** electrical measurement.

The performance of DTA studies included in the meta-analysis, in terms of specificity and sensitivity, were evaluated based on the confusion matrix (filled with the TP, FN, TN, FP values reported by the investigated study) and visually compared using SROC curves (Figure 3). Different studies are grouped and depicted based on the technique implemented. See Supplementary Figures 5–7 for further details on TN, TP, FN, FP values and 95%CI of each study. Also, DTA studies excluded from the meta-analysis were reported for completeness. The aforementioned figures also showed a forest plot for each technique.

The results of the meta-analysis performed suggests that RCM studies generally report high sensitivity (88.2%, 95%CI 80.3–93.1%) paired with high specificity (65.2%, 95% CI: 55.0–74.2%). Exceptions to this high performance were found in Pampena et al. (28) were the Segura algorithm, with threshold ≥−1 for melanoma diagnosis, reached 92% sensitivity but 30% specificity, while a sensitivity of 81% and a specificity of 51% was achieved with Pellacani-2012 scoring system. Stanganelli et al. (23) reported the largest range in terms of 95% CI.

MI generally presented high sensitivity (93%, 95%CI 75.3–98.3%) and specificity (71.2%, 95% CI 17.6–96.6%) in melanoma vs. other benign skin lesions classification tasks.

The only DTA study exploring OCT for melanoma diagnosis reported 92.4% specificity and 74.1% sensitivity with a 95% CI of (83–97%) and (54–89%), respectively; as only a study reported the use of OCT in clinical setting for melanoma diagnosis, the technique was excluded from meta-analysis, as a single study was not enough to validate the technique and SROC analysis could not be applied.

Overall, spectroscopy presented high sensitivity (93.0%, 95% CI: 92.8–93.2%), and high specificity (85.2%, 95% CI: 84.9–85.5%) in melanoma classification.

EIS studies generally presented high sensitivity (95%, 95% CI: 88.9–97.8%) but low specificity (48.9%, 95% CI: 30.5–67.6%). Ceder et al. (59) and Rocha et al. (60) reported a specificity of 71% and 83%, respectively, employing the same technique in melanoma recognition. Recent studies (63, 65) did not report TP, TN, FP, FN values and performance could not be analyzed. Ceder et al. (59), with follow-up at 3 months, presented a sensitivity of 100% for a 70% specificity but the 95% CI were 3–100% and 48–89%, respectively.

None citations employing thermal measurements for melanoma diagnosis reported TN, TP, FP, FN values, thus, performances in terms of specificity and sensitivity in melanoma diagnosis of thermal technique were not analyzed (i.e., studies were not included in the meta-analysis and in the forest plot, in the Supplementary Figure 7, were depicted using red lines).

## Discussion

The aim of this review is to compare the diagnostic performance of non-invasive techniques (Figure 2) in combination with or as an alternative to dermoscopy for melanoma detection. A comprehensive literature review yielded 62 results. Of those studies, 38 evaluated the diagnostic performance of a specific technique and were included in the Quadas-2 analysis, of which 29 were included in the meta-analysis (i.e., SROC evaluation highlighting Quadas-2 results, as described Methodological analysis).

Comparing SROC curves (Figure 3), optical spectroscopy achieved the best diagnostic performance in terms of specificity (85.2%) and sensitivity (93%) among all the investigated techniques in melanoma diagnosis (Figure 3C). Only three studies reported the use of this technique for the task of interest (i.e., diagnosis of melanoma vs. benign lesions) and were characterized by wide CIs of specificity and sensitivity. RCM technique instead, was used by several studies, where both sensitivity and specificity CIs are the smallest across all techniques included in this review. Moreover, Alarcon et al. (22) achieved the highest diagnostic performance among all techniques, maximizing specificity and sensitivity (92% and 98%, respectively) with a narrower CI (9% and 7%, respectively) when pairing RCM with dermoscopy. In general, most studies maximized sensitivity with respect to specificity. Moreover, all diagnostic non-invasive techniques, except OCT, reported lower values for specificity than sensitivity. The need to achieve higher sensitivity is intrinsic in a cancer screening procedure as a misdiagnosis of a malignant lesion negatively affects patients' prognosis. OCT could not be considered the best implemented technique as only one study was found and included in the QUADAS 2-tool analysis, hence inclusion in the meta-analysis was not possible.

As it could be seen from QUADAS-2 quality assessment summary reported in Supplementary Figures 1–4, the majority of studies (including the RCM ones) scored high overall risk of bias and concerns regarding applicability, decreasing overall results robustness. Many of the analyzed studies chose an *ad hoc* threshold to maximize sensitivity (e.g., Bodén et al. (33), Godoy et al. (73)) unbalancing the classification output and skewing the performance of the classifier in a biased way. Moreover, this threshold was specified after the diagnostic task (as described in the Results section), biasing the final test results as reported in the index test domain (Supplementary Figures 2, 4). In some cases, the diagnosis was performed by automatic classification algorithms. These were mostly simple machine learning algorithms (19, 33, 40, 41, 50, 52, 55–57, 61, 72–74, 76, 77). Other studies (44, 46, 49, 54, 56) implemented artificial neural networks, however, scarce details on layers, hyperparameters and training regimen were reported hampering reproducibility. Among these 19 studies, the datasets used were usually limited in terms of sample size, i.e., median 137, mean amplitude deviant ±292. Almost 63% of the studies had a dataset with a sample size lower than 200 samples, possibly limiting the performance of the implemented classification algorithms. Four studies (19, 33, 50, 55) implemented a leave-one-out cross validation due to their small sample size. The data splitting strategy in training and test set was not properly reported in three studies (40, 41, 46). In few cases, some data included in the training phase were also used as part of the test set (52, 57), biasing the reported performance. In other cases (44, 74), the datasets were equally split in training and test set. Some studies (57, 73, 74, 76, 77) used features extracted from the original data to feed their algorithms. Although this operation could reduce computational cost, the resulting classification performance could be affected since the features extracted manually might not represent most of the information content of the original dataset. The classification performances of the algorithms belonging to different studies cannot be directly compared since different classification tasks were implemented. Most studies aimed to distinguish MM lesions from benign lesions and thus, were included in the meta-analysis as this is the clinically relevant scenario to which the focus of this review is addressed. Four studies were excluded from our meta-analysis due to the different classification tasks implemented (i.e., malignant vs. benign or melanoma vs. “skin”). Rodriguez Diaz et al. (54) evaluated elastic scattering spectroscopy diagnostic performance in malignant lesions detection against benign lesions in a dataset composed of 357 lesions, of which 126 malignant (14 MM and 112 non-melanoma skin cancers). The achieved performances were 94% (89–98% CI) sensitivity and 36% (30–43% CI) specificity. Although, the number of TP is high (119 with respect to 126, i.e., the total malignant lesions), there are few samples related to melanoma type, thus, this performance cannot be compared accurately with the other techniques presented in this review. Sgouros et al. (42) used the MI technique to distinguish malignant lesions (31, of which 18 MM, 10 basal cell carcinomas and 3 squamous cell carcinoma) from benign skin lesions (157). The used algorithm achieved 84% (66–95% CI) sensitivity and 46% (19–75% CI) specificity when the outcome was compared with histopathological results while an 86% (57–95% CI) sensitivity and a 65% (57–73% CI) specificity when compared with dermoscopy. A similar approach was implemented by Delpueyo et al. (44), reaching a sensitivity of 91% (82–97% CI) and specificity of 54% (46–63% CI) using a lesion dataset of 95 MM, 44 basal cell carcinomas and 290 banal nevi. Although, the number of MM is higher in these two studies with respect to the Rodriguez Diaz one, including other type of skin cancers could impair the final evaluation of the techniques' performance in the detection of MM with respect to benign lesions. Eventually, Shirkavand et al. (52) aimed to distinguish MM from healthy skin using elastic scattering spectroscopy, reaching a good performance in terms of sensitivity (80%, 56–94% CI) and specificity (95%, 75–99% CI). The achieved specificity is the highest reached among all spectroscopic techniques. Nevertheless, no information was collected in the detection of MM with respect to other skin lesions (that represents the clinical scenario investigated by this review).

The QUADAS-2 analysis of the included studies concluded that the risk of including biases in experimental protocols and patient's selection is high among all the investigated diagnostic techniques (Supplementary Figures 1–4). The most common bias shared among studies is a lack or sub-optimal participant recruitment procedure. Some studies (33, 40, 72, 73) aimed to maximize performance metric specifying diagnostic threshold after the diagnostic task itself, introducing a significant bias into the index test domain. Inclusion and exclusion criteria in participant selection are not standardized and unclear or missing in 30 studies out of 38. The inclusion of only dermatological pre-selected lesions in all studies except six (19, 28, 38, 44, 50, 58), leaded to a high incidence melanoma setting and made extrapolation of performance results, to a primary care setting, challenging. Half of the studies, evaluating RCM, are retrospective analysis (Table 1). One of the main characteristics of retrospective studies is that the lesions were already targeted and treated at the time in which the study was carried on, hence an operator misdiagnosis has no consequences on the patient outcomes. The lack of responsibility in missing a melanoma may lead to higher specificity than the one achieved in an earlier clinical scenario.

The diagnostic performance evaluated and compared in the review did not take into account the integration of anamnestic information in the diagnostic process, due to the absence of those data in all the evaluated papers, even if those information might have some effect on the final diagnostic performance. Hence, it is currently difficult to quantify the contribution of those information in the diagnostic process itself.

RCM and OCT are considered to provide an *in-vivo* “virtual biopsy” of the lesion. Since RCM enables the visualization of characteristic features with cell-level resolution (such as, honeycomb pattern and pagetoid cells), it may be adopted especially in those clinical scenarios where a difficult to diagnose lesion is examined, as with lentigo maligna melanoma vs. benign macules of the face (87–91). The scoring systems of these techniques are based on features recognition that are then analyzed by an expert user to attain an accurate diagnosis. Hence, these scoring systems are user-dependent, and the informative content of the images may not be completely exploited by visual examination. Both RCM and OCT required a reconstruction following a mosaic like composition techniques that merges several images depicting a small part of a lesion. In fact, this approach is characterized by instruments with a small field of view. This characteristic and the associated reconstruction procedure might lead to artifacts and misalignments. The initial cost of these instruments and the time required to achieve a diagnosis are higher when compared to homologous metrics recorded using dermoscopy. More recent studies (44, 46), concerning MI, reported the use of arrays of LEDs illumination systems that shows promising characteristics as this kind of system can measure biochemical information with high spatial resolution while reducing instrument dimensions, costs and acquisition time. These studies (44, 46) reported preliminary results on melanoma-nevi differentiation, unfortunately counting no clinical trials study yet.

Spectroscopy, such as, MI, employs different wavelengths to detect biochemical information (e.g., hemoglobin and melanin content) on a single point-like spot, thus, several measurements are needed. Currently, neither an optimal experimental design nor a standardization among setups for spectroscopic measurements has been defined. Moreover, basic validation studies to identify spectral features and relative histopathological correlates are needed to define a robust and/or interpretable scoring system. Given these characteristics and hence the relative complexity in interpreting spectral features, all the current approaches used automatic algorithms to classify spectra and reduce output variability. *DermaSensor*™ achieved 100% sensitivity in the detection of MM, but the tool showed low specificity, i.e., 36%, possibly leading to a rise the number of unnecessary biopsies to provide support for the diagnosis of ambiguous lesions.

While the correlation between optical-based techniques and histological features is well-validated in literature, the biological correlation with EIS measurements is still unclear. EIS studies employed the commercially-available *Nevisense* with a dedicated scoring system (57, 58). However, it is unclear how this score is assigned to the investigated lesion, furthermore, most of the misdiagnosis were done on early-stage melanomas (58, 63). A limitation of this technique is the need to take multiple measures of the same lesion, as the instrument's electrodes area does not cover the entire lesion.

Studies involving thermal measurements show mainly preliminary and qualitative results. Thermal images of the entire area can be acquired without skin contact and in <5 min (66, 67, 69, 70, 72, 73). The diagnostic performance of this technique is still unclear since few studies (69, 72–74, 76) used the technology with the aim of making a diagnosis, moreover the results reported were not exhaustively detailed from a methodological point of view. Further studies are needed to uncover the histopathological underpinnings on which this system acts. System integration was not considered except for Okabe et al. (75) where the external thermal stress and the measurement sensors were integrated in a single pen-shaped device.

## Conclusions

This review reports the diagnostic performances of available non-invasive techniques alternative to dermoscopy for the diagnosis of skin melanoma. Overall, optical spectroscopy scored the highest diagnostic performances (average sensitivity and specificity, 93% and 82.2%, respectively, see Figure 3). Although, only three studies reported the performance metrics in the diagnostic task analyzed, leaving possible concerns about the robustness and variability associated with these metrics. MI achieved high diagnostic performance (average sensitivity and specificity, 93% and 71.2%, respectively, computed using only four studies) but reported the widest CIs range (17.6–96.6% for specificity and 75.3–98.3% for sensitivity). EIS, evaluated on five studies, achieved 95% average sensitivity paired with the lowest average specificity among the investigated techniques (48.9%), which also reported a wide CI (30.5–67.6%). RCM performances, instead, was computed analyzing six different studies, of which one compared six diagnostic algorithms (average sensitivity and specificity, 88.2% and 65.2%, respectively) and displayed also small 95% CIs, 80.3–93.1% and 55–74.2%, respectively. Moreover, RCM scored the highest performance when paired with dermoscopy (Alarcon et al. (22) sensitivity 98%, 95% CI 92%–99%; and specificity 92%, 87–96%; see Supplementary Figure 5) and thus, exceeding dermoscopy-alone diagnostic performance (8). Analyzing SROC curves, highlighted the presence of relatively wide sensitivity and specificity CIs across all the techniques (especially optical spectroscopy and multispectral imaging), rising concerns about the reliability of reported performances. Regarding the QUADAS evaluation, 84.2% of studies were classified at high risk of bias and 63.2% had applicability concerns.

**Figure 4:**
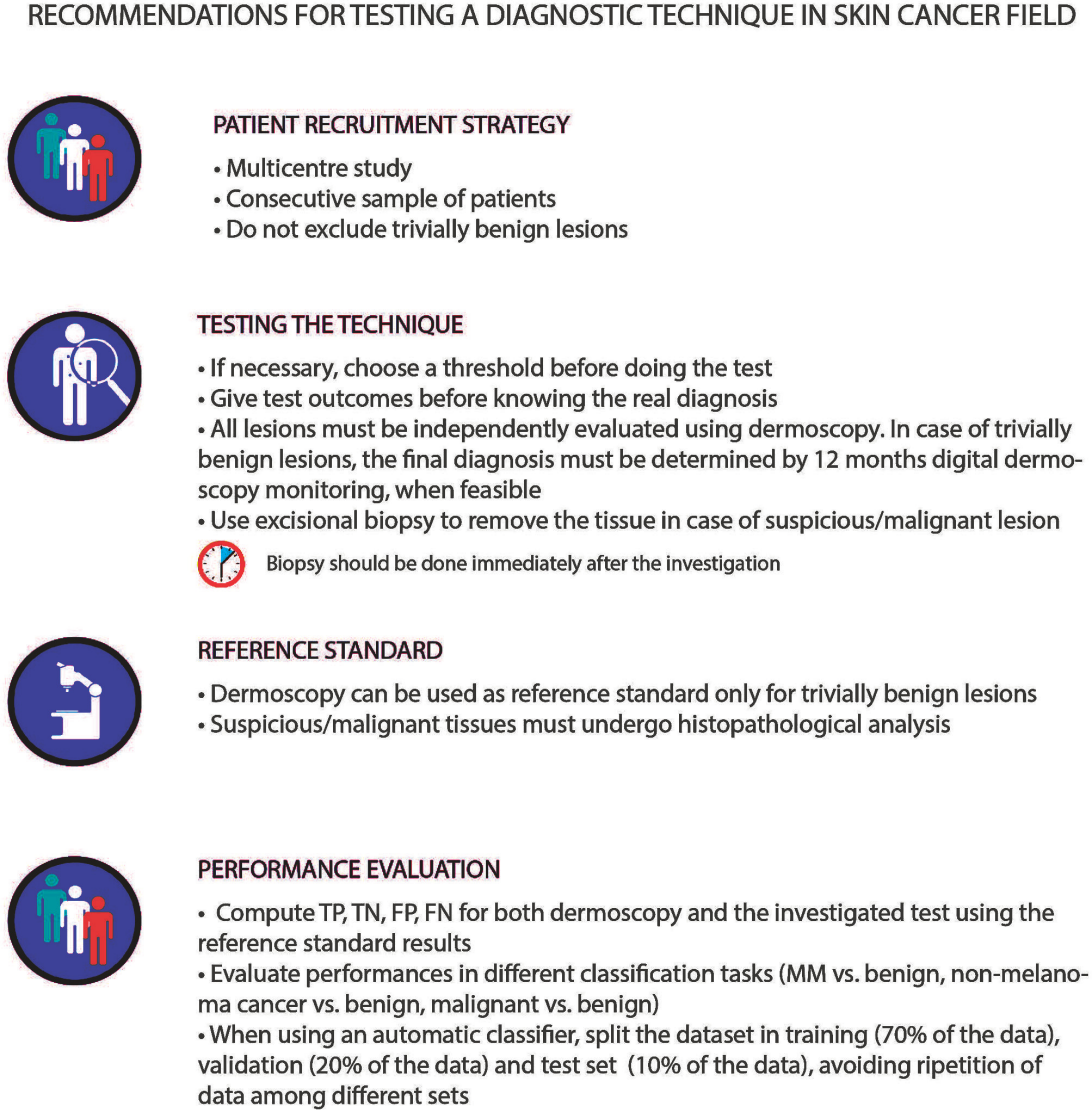
Best practice in assessing techniques performances within the dermatological field. The first three guidelines were proposed based on the QUADAS-2 tool requirements, while the last one was derived by the literature review. The lesions chosen for the investigation should belong to a study population that reflects the standard population. The outcomes of a technique should be compared to the histopathological analysis of the lesion itself, except for trivially benign lesions, (in this case, dermoscopy can be used as an alternative). Indeed, histopathology is the current reference gold standard in this field, even if with its own limitations. As described in literature (86) the failure rate of histopathological analysis depends on the type of biopsy involved. Thus, excisional biopsy is advised. This approach stems from common clinical practice, albeit it may introduce possible biases in the classification trustworthiness of this type of lesions. It is known that the use of different reference standard for different lesion types may hamper the final evaluation of the performances of each technique, as well as the comparison with dermoscopy itself. The proposed dataset splitting is one of the main splitting methods used in this field, however, there can be others suitable for the specific task under investigation. MM, malignant melanoma; TP, True Positive; TN, True Negative; FP, False Positive; FN, False Negative.

Beyond the reported metrics, other unmeasurable but crucial factors, such as, technique usability, ease of use, results interpretability, and clinical acceptance, may hamper the adoption and clinical usage of a technique. Meta-analytical evidence, stemming from the analysis of the literature provided in this review, may be used as a quantitative and methodologically sound support for the selection of the most suitable technique for a specific clinical case, timing or workflow, considering always the clinician at the center of the decision process. The most relevant limiting factors that precluded a systematic comparison of all the presented techniques were (i) heterogeneity in the type of studies implemented (e.g., retrospective analysis, clinical trials); (ii) differences in testing strategy (as highlighted by the QUADAS analysis); and (iii) the definition of the diagnostic tasks (e.g., melanoma vs. nevus or benign vs. malignant). These methodological biases may affect results and invalidate performance comparison. Given these limitations, future studies, addressing the performance evaluation of an alternative technique to dermoscopy for melanoma diagnosis, may benefit from following best practice recommendations, as those suggested in Figure 4. These suggestions are tailored to better validate and compare the diagnostic performance of the investigated technique and should always be applied favoring patient protection over any other circumstance. In addition, the aforementioned best practices are not designed to be adopted as common clinical practice.

Moving further, some of the included techniques (e.g., RCM) are extensively validated in literature but their usage within the clinical setting is still limited due to their high costs and low clinical acceptance. To widen the adoption of those techniques, a significant effort should be done to increase technology accessibility, mainly reducing the overall costs and expertise needed to use those technologies. Moreover, to maximize reproducibility, an optimal diagnostic technique should: (i) acquire data in a short period of time (e.g., minute or less), ultimately limiting artifact induced by patient's movements; and (ii) minimize errors induced by operators due to suboptimal data acquisition or erroneous subjective evaluation of gathered data. Finally, to increase clinical acceptance and adoption of new solutions, the ideal technology should display a balanced trade-off between diagnostic accuracy and overall complexity of use. Indeed, the ideal technique should provide objective information related to a well-known biological correlate in an easy-to-understand manner for the clinician.

## Data Availability Statement

The raw data supporting the conclusions of this article will be made available by the authors, without undue reservation.

## Author Contributions

AB, AC, and TB contributed in the conception and design of the study. AB and AC performed the literature review, data extraction, statistical analysis, and wrote the first draft of the manuscript. GC and TB contributed to the writing of the manuscript and supervised the entire research effort. All authors contributed to the article and approved the submitted version.

## Conflict of Interest

This work was carried on in the framework of a joint project (Advanced Laboratory Automation) between Scuola Superiore Sant'Anna and Inpeco SA, which was funded by the latter. The authors declare that the research was conducted in the absence of any commercial or financial relationships that could be construed as a potential conflict of interest.

## Supplementary Material

Supplementary Figure 1 QUADAS-2 tool analysis of bias and applicability of optical imaging techniques. Nineteen studies were included in optical imaging, of which 8 for RCM, 1 for OCT, and 10 for multispectral. The Index test is the diagnostic test that is evaluated against a reference standard test (dermoscopy or histopathology) in a study of test accuracy. The risk of bias of the reference standard was considered low since both dermoscopy and histopathology are well-validated in literature, however, those studies using different reference standards for different types of lesions were scored with high risk of bias in flow and timing. **(A)** Table form reporting scoring for each domain for optical imaging DTA studies. **(B)** Proportion of optical imaging studies with low, high, unclear risk of bias. Number of studies is reported on the graph. **(C)** Proportion of optical imaging studies with low, high, unclear concerns regarding applicability. Number of studies is reported on the graph.

Supplementary Figure 2 QUADAS-2 tool analysis of bias and applicability of optical spectroscopy techniques. Seven studies were included in the analysis. The Index test is the diagnostic test that is evaluated against a reference standard test (dermoscopy or histopathology) in a study of test accuracy. The risk of bias of the reference standard was considered low since both dermoscopy and histopathology are well-validated in literature, however, those studies using different reference standards for different types of lesions were scored with high risk of bias in flow and timing. **(A)** Table form reporting scoring for each domain for optical spectroscopy DTA studies. **(B)** Proportion of optical spectroscopy studies with low, high, unclear risk of bias. Number of studies is reported on the graph. **(C)** Proportion of optical spectroscopy studies with low, high, unclear concerns regarding applicability. Number of studies is reported on the graph.

Supplementary Figure 3 QUADAS-2 tool analysis of bias and applicability of electrical skin impedance (EIS) techniques. Seven studies were included. The Index test is the diagnostic test that is evaluated against a reference standard test (dermoscopy or histopathology) in a study of test accuracy. The risk of bias of the reference standard was considered low since both dermoscopy and histopathology are well-validated in literature, however, those studies using different reference standards for different types of lesions were scored with high risk of bias in flow and timing. **(A)** Table form reporting scoring for each domain for EIS DTA studies. **(B)** Proportion of EIS studies with low, high, unclear risk of bias. Number of studies is reported on the graph. **(C)** Proportion of EIS studies with low, high, unclear concerns regarding applicability. Number of studies is reported on the graph.

Supplementary Figure 4 QUADAS-2 tool analysis of bias and applicability of thermal measurements techniques. Five studies were included. The Index test is the diagnostic test that is evaluated against a reference standard test (dermoscopy or histopathology) in a study of test accuracy. The risk of bias of the reference standard was considered low since both dermoscopy and histopathology are well-validated in literature, however, those studies using different reference standards for different types of lesions were scored with high risk of bias in flow and timing. **(A)** Table form reporting scoring for each domain for thermal DTA studies. **(B)** Proportion of thermal studies with low, high, unclear risk of bias. Number of studies is reported on the graph. **(C)** Proportion of thermal studies with low, high, unclear concerns regarding applicability. Number of studies is reported on the graph.

Supplementary Figure 5 Forest plot of specificity and sensitivity of techniques investigated in those studies analyzed using the QUADAS-2 tool for optical based techniques (both imaging and spectroscopy). These metrics were evaluated based on the reported values of True Positive (TP), False Negative (FN), True Negative (TN), and False Positive (FP). Sensitivity and specificity were depicted with the corresponding 95% confidence interval calculated using the Clopper-Pearson method. A red dashed line in the Forest Plot represents studies analyzed using the QUADAS-2 that did not explicit TP, FN, TN, FP values. Studies were listed based on the technique exploited: Reflectance confocal microscopy (RCM, a-b), Multispectral imaging (MULTISP, c-d), Optical Coherence Tomography (OCT, e-f), Spectroscopy (SPECT, g-h). The same study is listed more than once if different thresholds, classification tasks or timing were reported in the study. For Pampena et al. (28) seven rows were reported indicating the algorithm tested (i.e., Pellacani 2005, Segura 2009, Pellacani 2021, Borsari 2018 and Borsari 2018 using only RCM) and the threshold used within the algorithm is reported in round brackets. In multispectral imaging, for Sgouros et al. (42) two different reference standards were evaluated and reported: Histology and dermoscopy. For Garcia-Urbe et al. (49) only the performance achieved in melanoma vs. benign pigmented lesions was reported in the graph.

Supplementary Figure 6 Forest plot of specificity and sensitivity of techniques investigated in those studies analyzed using the QUADAS-2 tool for skin electrical measurements techniques (EIS). These metrics were evaluated based on the reported values of True Positive (TP), False Negative (FN), True Negative (TN), and False Positive (FP). Sensitivity and specificity were depicted with the corresponding 95% confidence interval calculated using the Clopper-Pearson method. A red dashed line in the Forest Plot represents studies analyzed using the QUADAS-2 that did not explicit TP, FN, TN, FP values. The same study is listed more than once if different thresholds, classification tasks or timing were reported in the study. In particular, Rocha et al. (60), *t* = 0 is referred to the diagnosis done at the first time the clinician had seen the patient, while *t* = 3 is referred to results obtained during the 3 months follow up. Ceder et al. (59) *t* = 3 is referred to results obtain by the authors during the follow up. Mohr et al. (57) and Malvehy et al. (58) instead achieved two performances using different classification tasks (melanoma: melanomas vs. benign lesions, all: all malignant lesions vs. benign lesions).

Supplementary Figure 7 Forest plot of specificity and sensitivity of techniques investigated in those studies analyzed using the QUADAS-2 tool for thermal measurements techniques (THERMAL). These metrics were evaluated based on the reported values of True Positive (TP), False Negative (FN), True Negative (TN), and False Positive (FP). Sensitivity and specificity were depicted with the corresponding 95% confidence interval calculated using the Clopper-Pearson method. A red dashed line in the Forest Plot represents studies analyzed using the QUADAS-2 that did not explicit TP, FN, TN, FP values.
