# Supplementary figures and images for: Comparative Analysis of Diagnostic Techniques for Melanoma Detection: A Systematic Review of Diagnostic Test Accuracy Studies and Meta-Analysis
Source: Front Med (Lausanne). 2021 Apr 21;8:637069. doi: 10.3389/fmed.2021.637069 (PMC8103840; doi:10.3389/fmed.2021.637069)

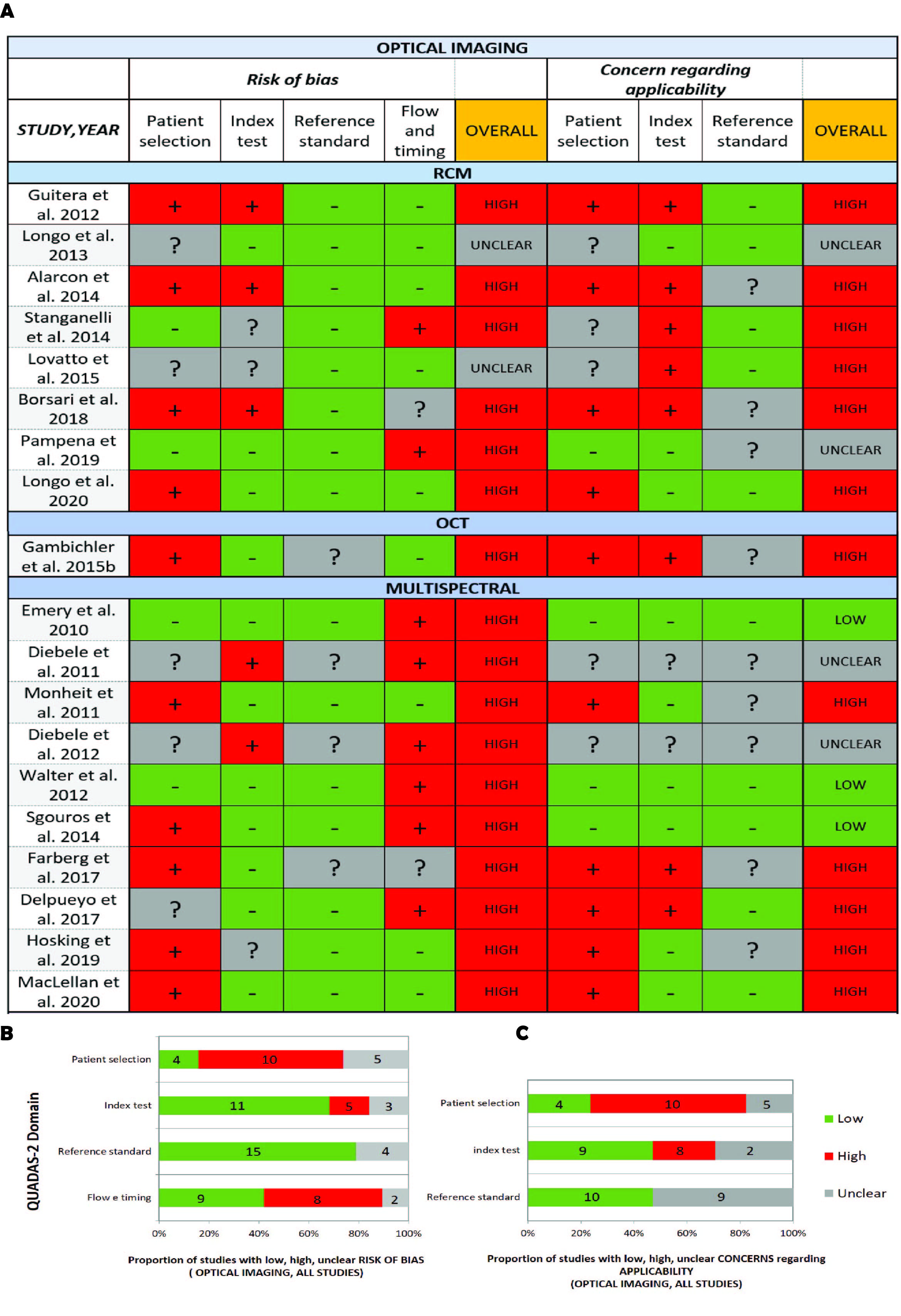

Supplement: Supplementary Figure 1 — QUADAS-2 tool analysis of bias and applicability of optical imaging techniques. Nineteen studies were included in optical imaging, of which 8 for RCM, 1 for OCT, and 10 for multispectral. The Index test is the diagnostic test that is evaluated against a reference standard test (dermoscopy or histopathology) in a study of test accuracy. The risk of bias of the reference standard was considered low since both dermoscopy and histopathology are well-validated in literature, however, those studies using different reference standards for different types of lesions were scored with high risk of bias in flow and timing. (A) Table form reporting scoring for each domain for optical imaging DTA studies. (B) Proportion of optical imaging studies with low, high, unclear risk of bias. Number of studies is reported on the graph. (C) Proportion of optical imaging studies with low, high, unclear concerns regarding applicability. Number of studies is reported on the graph. [file Image_1.jpg]

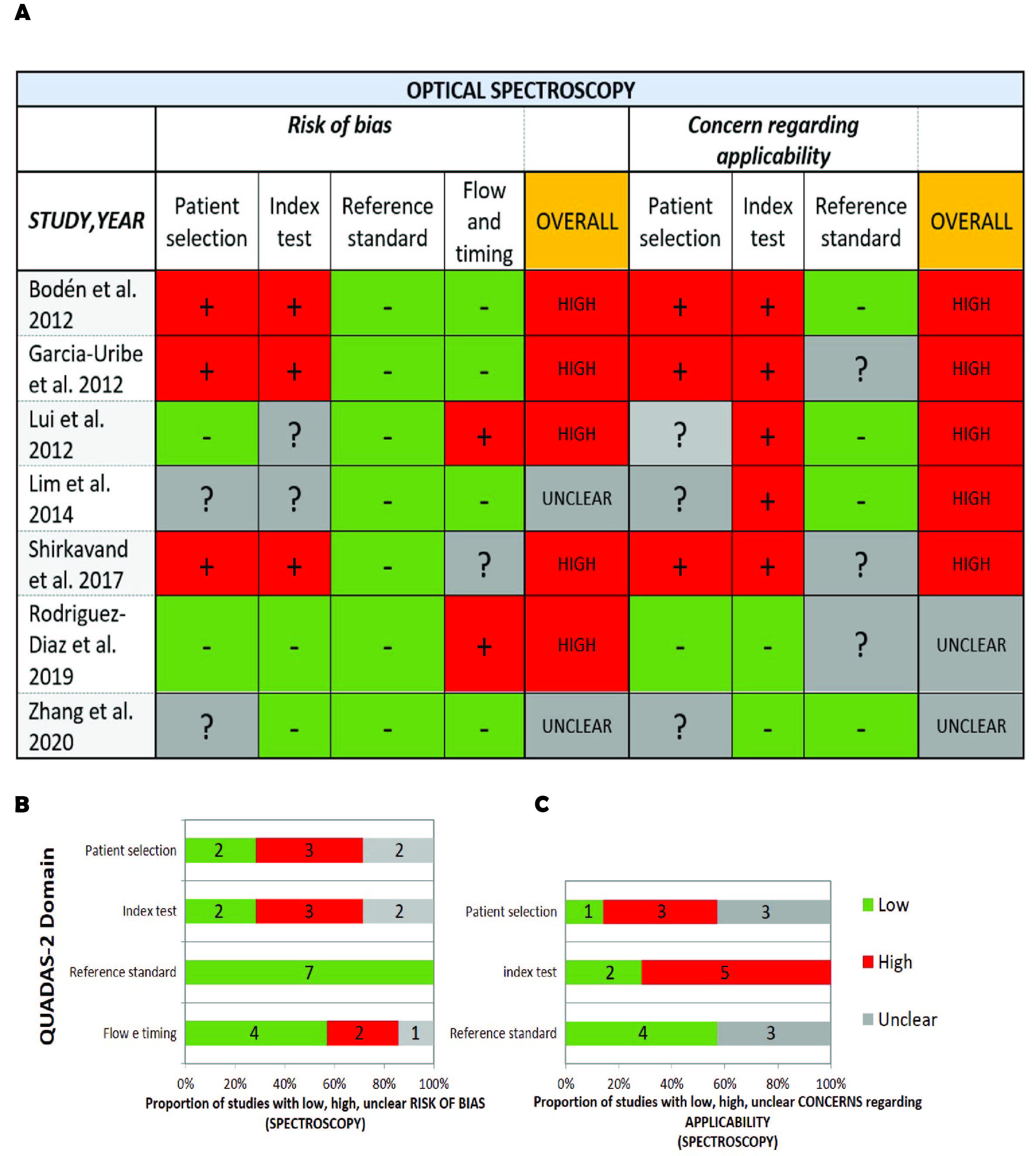

Supplement: Supplementary Figure 2 — QUADAS-2 tool analysis of bias and applicability of optical spectroscopy techniques. Seven studies were included in the analysis. The Index test is the diagnostic test that is evaluated against a reference standard test (dermoscopy or histopathology) in a study of test accuracy. The risk of bias of the reference standard was considered low since both dermoscopy and histopathology are well-validated in literature, however, those studies using different reference standards for different types of lesions were scored with high risk of bias in flow and timing. (A) Table form reporting scoring for each domain for optical spectroscopy DTA studies. (B) Proportion of optical spectroscopy studies with low, high, unclear risk of bias. Number of studies is reported on the graph. (C) Proportion of optical spectroscopy studies with low, high, unclear concerns regarding applicability. Number of studies is reported on the graph. [file Image_2.jpg]

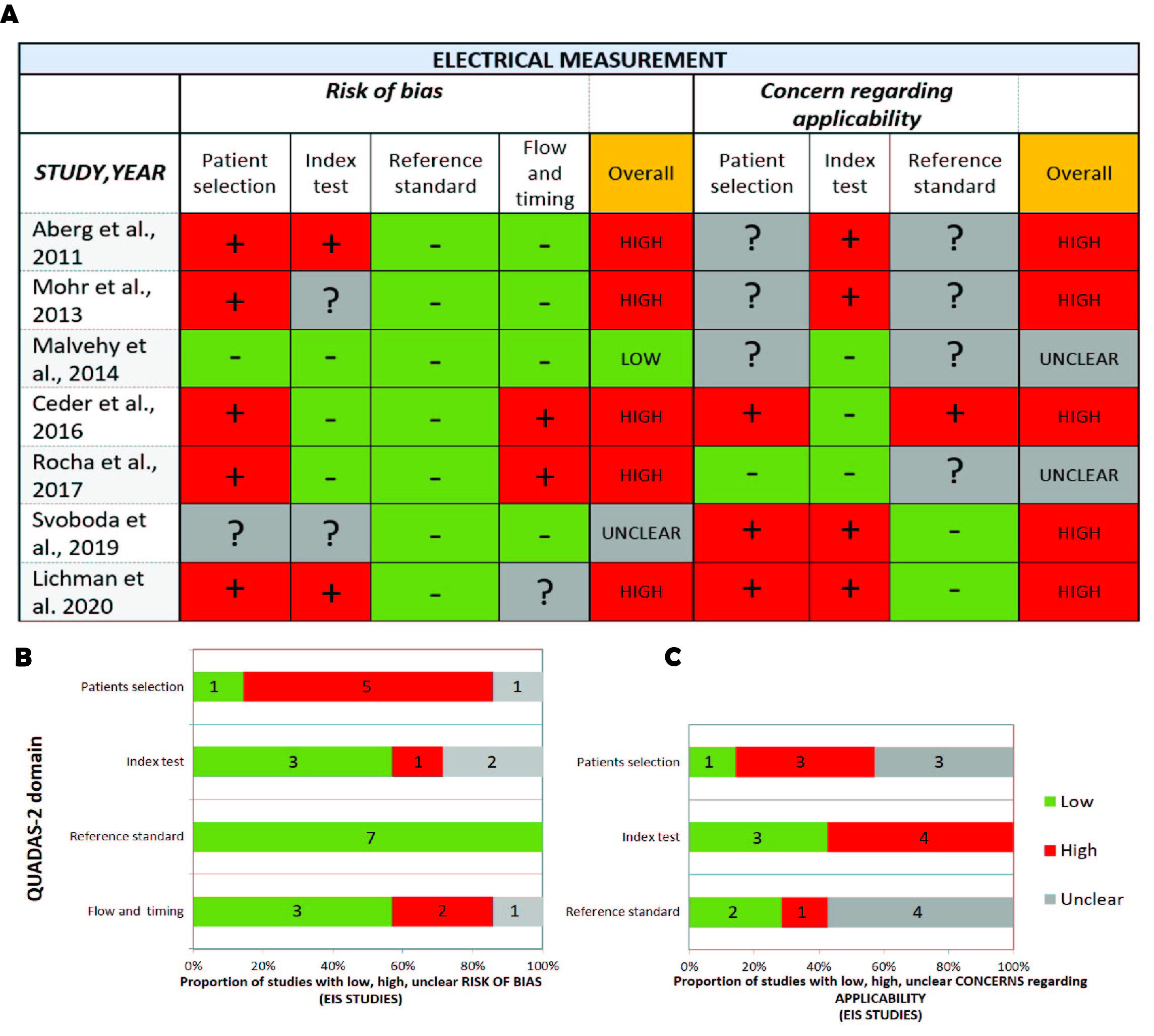

Supplement: Supplementary Figure 3 — QUADAS-2 tool analysis of bias and applicability of electrical skin impedance (EIS) techniques. Seven studies were included. The Index test is the diagnostic test that is evaluated against a reference standard test (dermoscopy or histopathology) in a study of test accuracy. The risk of bias of the reference standard was considered low since both dermoscopy and histopathology are well-validated in literature, however, those studies using different reference standards for different types of lesions were scored with high risk of bias in flow and timing. (A) Table form reporting scoring for each domain for EIS DTA studies. (B) Proportion of EIS studies with low, high, unclear risk of bias. Number of studies is reported on the graph. (C) Proportion of EIS studies with low, high, unclear concerns regarding applicability. Number of studies is reported on the graph. [file Image_3.jpg]

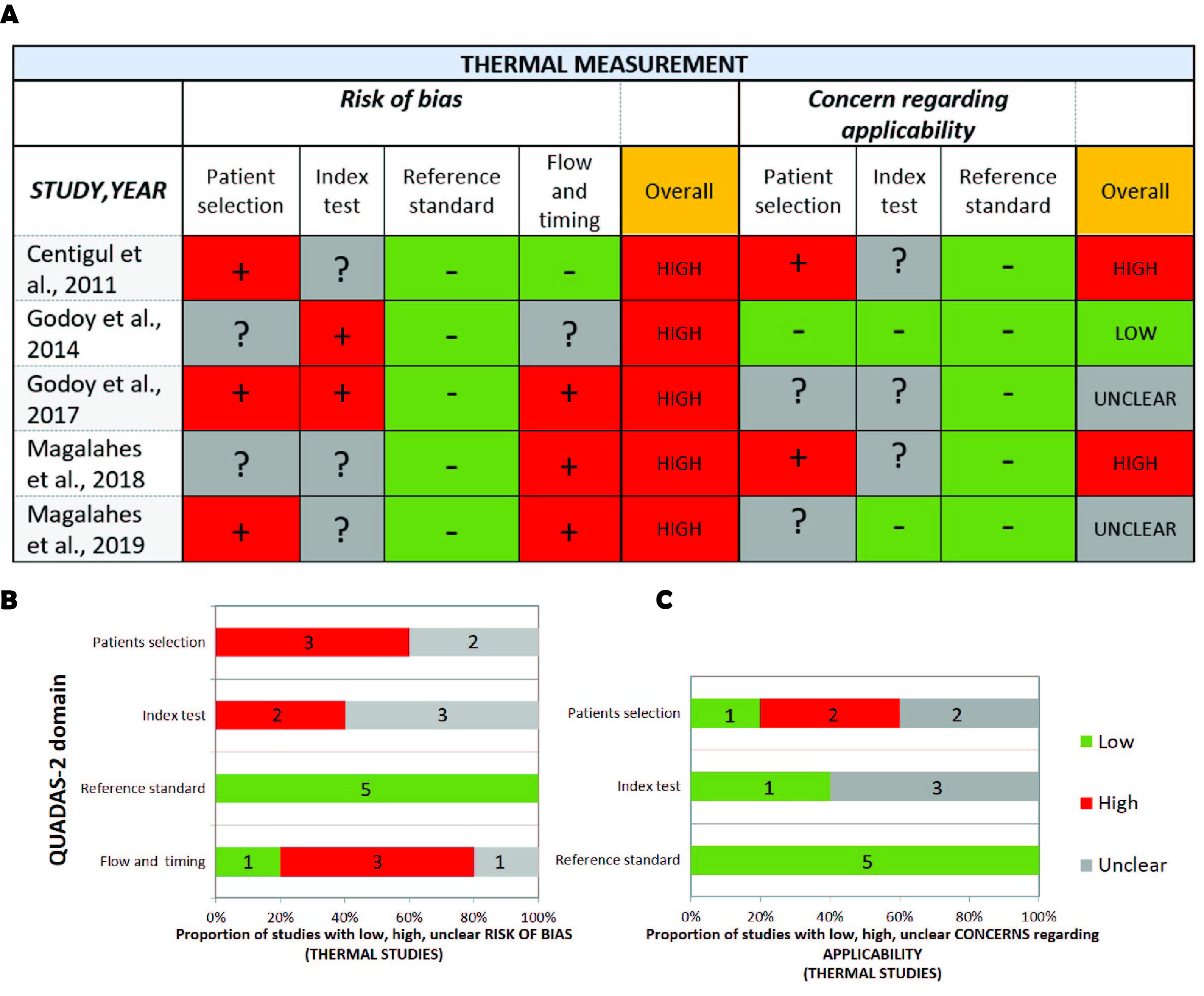

Supplement: Supplementary Figure 4 — QUADAS-2 tool analysis of bias and applicability of thermal measurements techniques. Five studies were included. The Index test is the diagnostic test that is evaluated against a reference standard test (dermoscopy or histopathology) in a study of test accuracy. The risk of bias of the reference standard was considered low since both dermoscopy and histopathology are well-validated in literature, however, those studies using different reference standards for different types of lesions were scored with high risk of bias in flow and timing. (A) Table form reporting scoring for each domain for thermal DTA studies. (B) Proportion of thermal studies with low, high, unclear risk of bias. Number of studies is reported on the graph. (C) Proportion of thermal studies with low, high, unclear concerns regarding applicability. Number of studies is reported on the graph. [file Image_4.jpg]

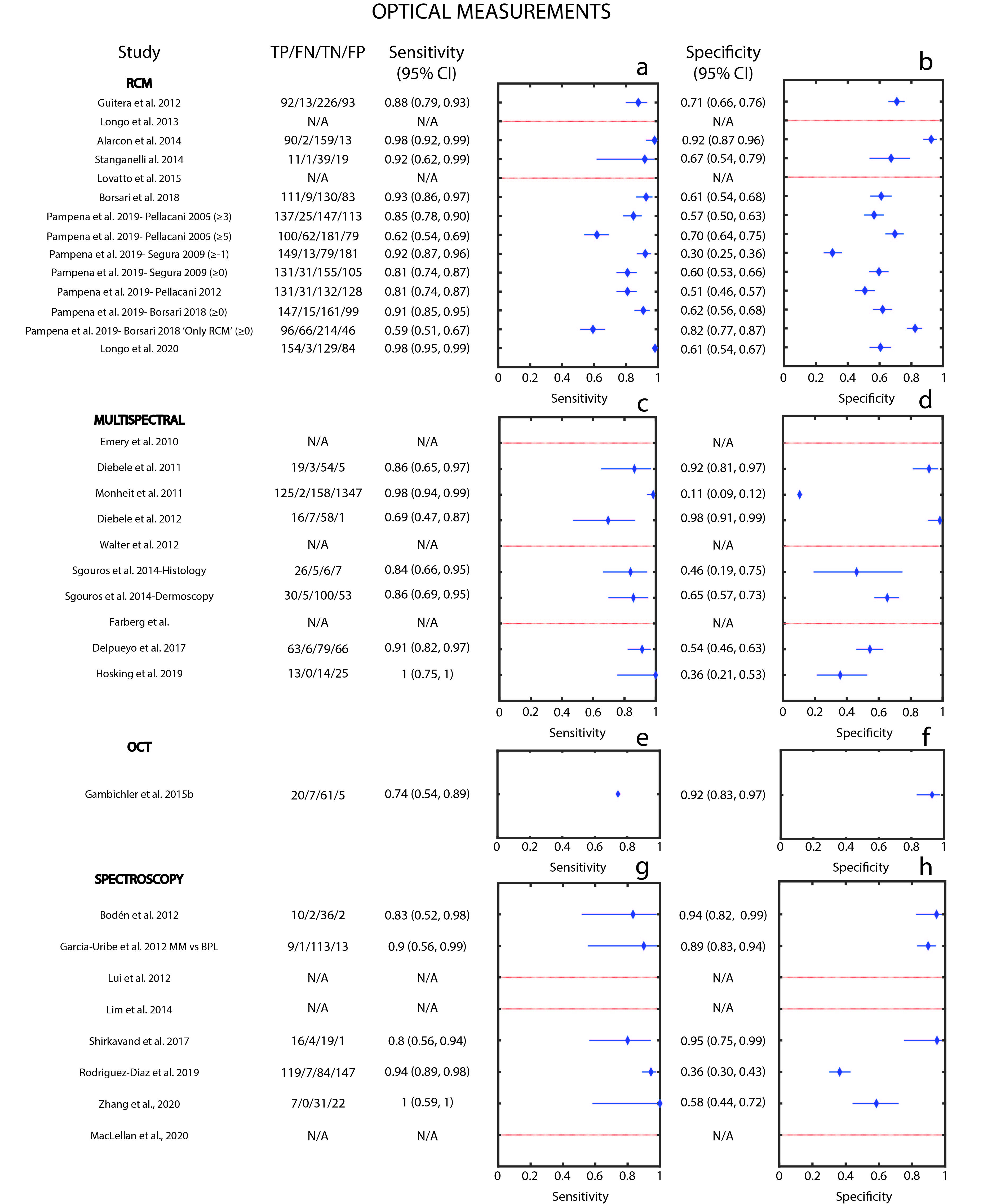

Supplement: Supplementary Figure 5 — Forest plot of specificity and sensitivity of techniques investigated in those studies analyzed using the QUADAS-2 tool for optical based techniques (both imaging and spectroscopy). These metrics were evaluated based on the reported values of True Positive (TP), False Negative (FN), True Negative (TN), and False Positive (FP). Sensitivity and specificity were depicted with the corresponding 95% confidence interval calculated using the Clopper-Pearson method. A red dashed line in the Forest Plot represents studies analyzed using the QUADAS-2 that did not explicit TP, FN, TN, FP values. Studies were listed based on the technique exploited: Reflectance confocal microscopy (RCM, a-b), Multispectral imaging (MULTISP, c-d), Optical Coherence Tomography (OCT, e-f), Spectroscopy (SPECT, g-h). The same study is listed more than once if different thresholds, classification tasks or timing were reported in the study. For Pampena et al. (28) seven rows were reported indicating the algorithm tested (i.e., Pellacani 2005, Segura 2009, Pellacani 2021, Borsari 2018 and Borsari 2018 using only RCM) and the threshold used within the algorithm is reported in round brackets. In multispectral imaging, for Sgouros et al. (42) two different reference standards were evaluated and reported: Histology and dermoscopy. For Garcia-Urbe et al. (49) only the performance achieved in melanoma vs. benign pigmented lesions was reported in the graph. [file Image_5.JPEG]

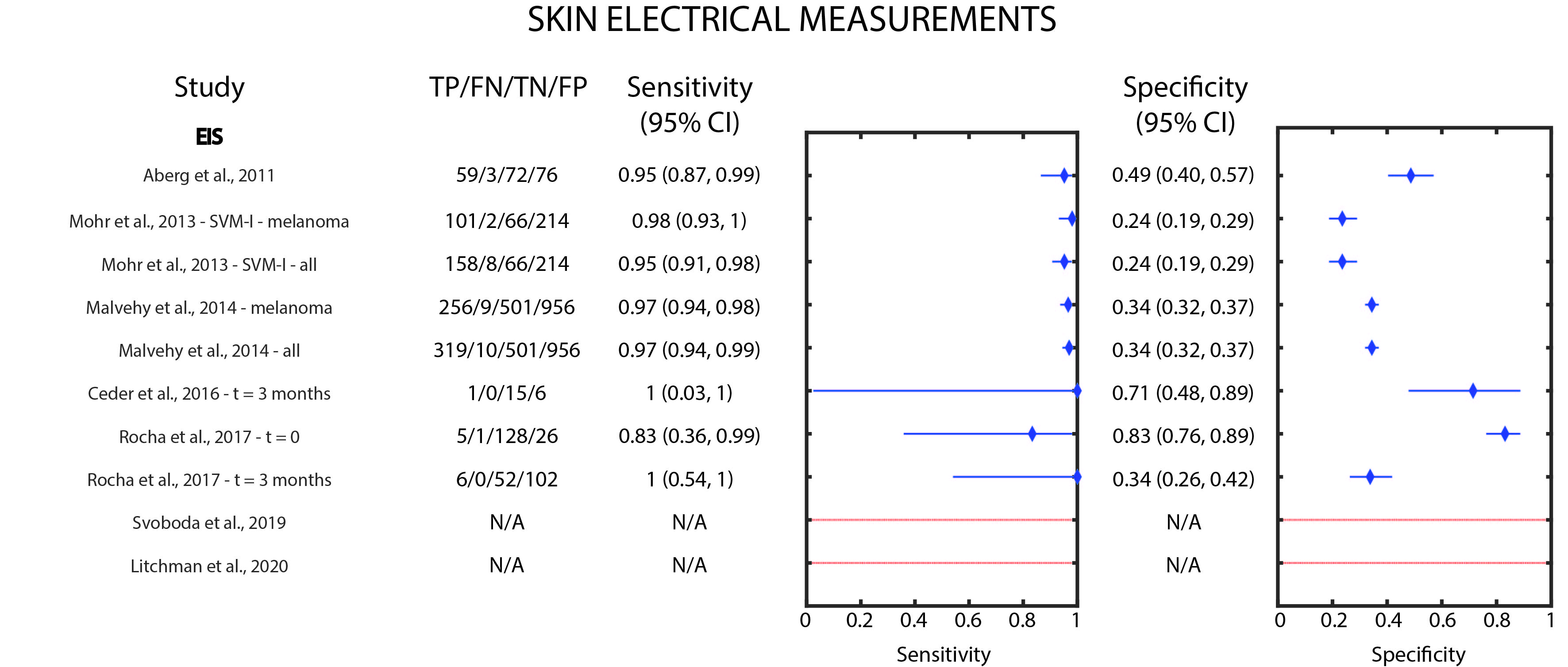

Supplement: Supplementary Figure 6 — Forest plot of specificity and sensitivity of techniques investigated in those studies analyzed using the QUADAS-2 tool for skin electrical measurements techniques (EIS). These metrics were evaluated based on the reported values of True Positive (TP), False Negative (FN), True Negative (TN), and False Positive (FP). Sensitivity and specificity were depicted with the corresponding 95% confidence interval calculated using the Clopper-Pearson method. A red dashed line in the Forest Plot represents studies analyzed using the QUADAS-2 that did not explicit TP, FN, TN, FP values. The same study is listed more than once if different thresholds, classification tasks or timing were reported in the study. In particular, Rocha et al. (60), t = 0 is referred to the diagnosis done at the first time the clinician had seen the patient, while t = 3 is referred to results obtained during the 3 months follow up. Ceder et al. (59) t = 3 is referred to results obtain by the authors during the follow up. Mohr et al. (57) and Malvehy et al. (58) instead achieved two performances using different classification tasks (melanoma: melanomas vs. benign lesions, all: all malignant lesions vs. benign lesions). [file Image_6.JPEG]

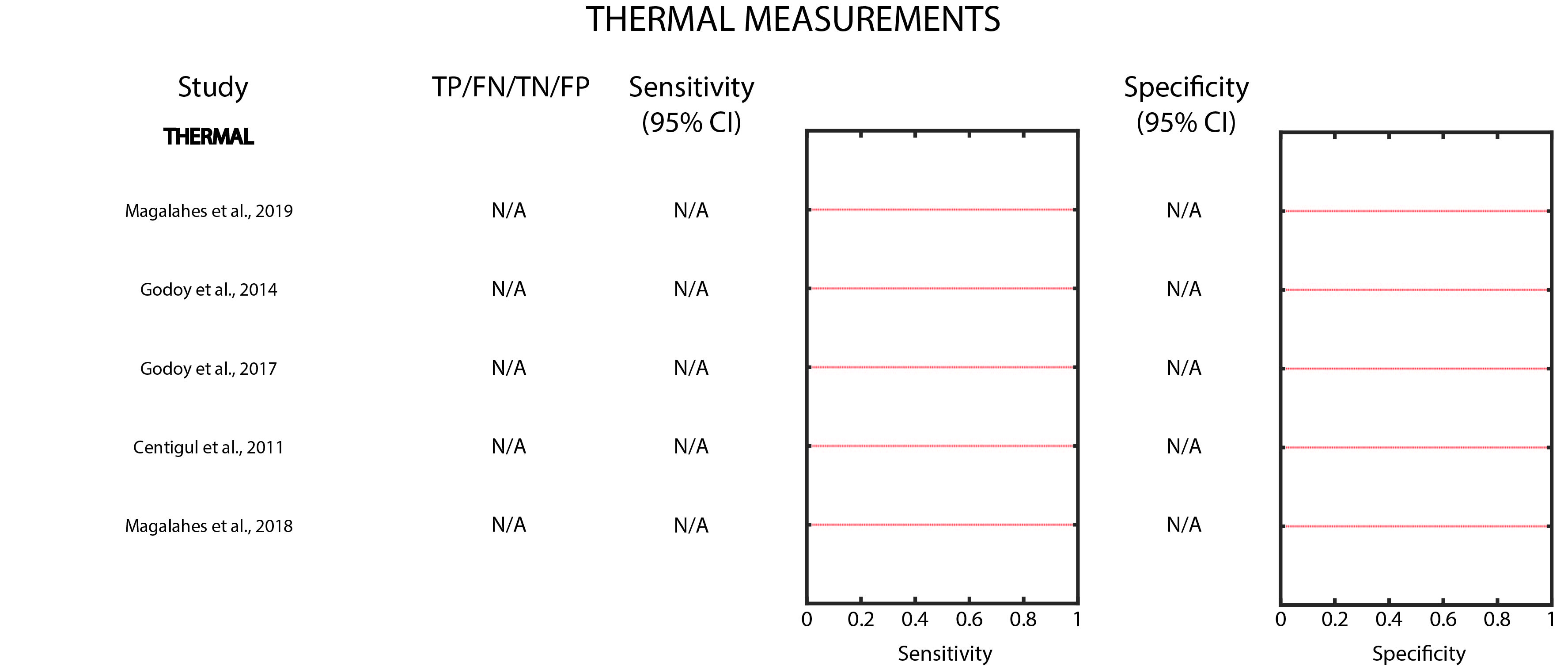

Supplement: Supplementary Figure 7 — Forest plot of specificity and sensitivity of techniques investigated in those studies analyzed using the QUADAS-2 tool for thermal measurements techniques (THERMAL). These metrics were evaluated based on the reported values of True Positive (TP), False Negative (FN), True Negative (TN), and False Positive (FP). Sensitivity and specificity were depicted with the corresponding 95% confidence interval calculated using the Clopper-Pearson method. A red dashed line in the Forest Plot represents studies analyzed using the QUADAS-2 that did not explicit TP, FN, TN, FP values. [file Image_7.JPEG]
